# Expression Patterns in Reductive Iron Assimilation and Functional Consequences during Phagocytosis of *Lichtheimia corymbifera*, an Emerging Cause of Mucormycosis

**DOI:** 10.3390/jof7040272

**Published:** 2021-04-03

**Authors:** Felicia Adelina Stanford, Nina Matthies, Zoltán Cseresnyés, Marc Thilo Figge, Mohamed I. Abdelwahab Hassan, Kerstin Voigt

**Affiliations:** 1 Jena Microbial Resource Collection, Leibniz Institute for Natural Product Research, and Infection Biology—Hans Knöll Institute (HKI), 07745 Jena, Germany; felicia.stanford@hki-jena.de (F.A.S.); ninamatthies@gmx.net (N.M.); mohamed.hassan@hki-jena.de (M.I.A.H.); 2 Institute of Microbiology, Friedrich Schiller University Jena, 07743 Jena, Germany; thilo.figge@hki-jena.de; 3 Applied Systems Biology, Leibniz Institute for Natural Product Research, and Infection Biology—Hans Knöll Institute, 12622 Jena, Germany; zoltan.cseresnyes@hki-jena.de; 4 National Research Centre, Pests & Plant Protection Department, 33rd El Buhouth St., Dokki, Giza 12622, Egypt

**Keywords:** host-pathogen interaction, heat-shock protein, virulence determinant, qRT-PCR, RNA, genome, phylogenetic tree, phenotype

## Abstract

Iron is an essential micronutrient for most organisms and fungi are no exception. Iron uptake by fungi is facilitated by receptor-mediated internalization of siderophores, heme and reductive iron assimilation (RIA). The RIA employs three protein groups: (i) the ferric reductases (Fre5 proteins), (ii) the multicopper ferroxidases (Fet3) and (iii) the high-affinity iron permeases (Ftr1). Phenotyping under different iron concentrations revealed detrimental effects on spore swelling and hyphal formation under iron depletion, but yeast-like morphology under iron excess. Since access to iron is limited during pathogenesis, pathogens are placed under stress due to nutrient limitations. To combat this, gene duplication and differential gene expression of key iron uptake genes are utilized to acquire iron against the deleterious effects of iron depletion. In the genome of the human pathogenic fungus *L. corymbifera*, three, four and three copies were identified for *FRE5*, *FTR1* and *FET3* genes, respectively. As in other fungi, *FET3* and *FTR1* are syntenic and co-expressed in *L. corymbifera*. Expression of *FRE5*, *FTR1* and *FET3* genes is highly up-regulated during iron limitation (Fe-), but lower during iron excess (Fe+). Fe- dependent upregulation of gene expression takes place in *LcFRE5* II and III, *LcFTR1* I and II, as well as *LcFET3* I and II suggesting a functional role in pathogenesis. The syntenic *LcFTR1* I–*LcFET3* I gene pair is co-expressed during germination, whereas *LcFTR1* II- *LcFET3* II is co-expressed during hyphal proliferation. *LcFTR1* I, II and IV were overexpressed in *Saccharomyces cerevisiae* to represent high and moderate expression of intracellular transport of Fe3+, respectively. Challenge of macrophages with the yeast mutants revealed no obvious role for *LcFTR1* I, but possible functions of *LcFTR1* II and IVs in recognition by macrophages. RIA expression pattern was used for a new model of interaction between *L. corymbifera* and macrophages.

## 1. Introduction

Mucorales are ubiquitous, thermotolerant fungi that can cause various types of infections summarized as mucormycosis. To date, 240 species have been classified in the order Mucorales, of which 20 species have been documented as the causative agents in the clinical setting [1,2,3]. *Rhizopus*, *Mucor* and *Lichtheimia* species account for over 70% of all mucormycosis cases and *Lichtheimia* species are usually the second most isolated in Europe [1,4,5,6]. To date, six species have been identified belonging to the genus *Lichtheimia*, three of which, *L. corymbifera*, *L. ramosa* and *L ornata* are common causes of nosocomial infections [7,8].

As typical environmental fungi, members of the Mucorales are non-pathogenic in immunocompetent individuals only causing rare allergic fungal sinusitis [9]. However, in the overtly immunocompromised such as patients with organ transplantation, patients of chronic steroids or immunosuppressive therapies, hematological or solid organ malignancies, mucoralean fungi can cause life threatening, debilitating disease associated with rapid progressive angioinvasion and extensive tissue necrosis [4,10,11,12,13,14]. Diabetics are also uniquely predisposed, particularly those patients with a history of diabetic ketoacidosis (DKA) [15,16,17]. As emerging pathogens, the causative agents of mucormycosis are notoriously difficult to diagnose and are resistant to most frontline antifungal therapies [15,16,17]. As such, the principal and most effective line of treatment remains aggressive surgical intervention to remove the necrotic tissue [17,18,19,20,21]. With surgical intervention, the mortality remains unacceptably high and new strategies for diagnosis and treatments are urgently required [22,23].

In the host, iron is essential for numerous physiological processes particularly in immune function and host immune response [24]. Iron promotes lymphocyte and macrophage differentiation, anti-microbial immune effector functions and immune cell metabolism [24,25,26]. As such, intracellular iron retention promoted by hepcidin, is highly important as it restricts iron availability to pathogens [27,28]. Intracellular iron trafficking is altered during infections depending on the location of the invading pathogen, i.e., intra or extracellular [24,29]. An important intracellular protein involved iron metabolism is ferritin [30]. Host ferritin is a large iron storage molecule consisting of two subunits: H-ferritin (Fth) and L-ferritin (Ftl) and is not only an iron reservoir but it is known to protect cells from the effects of free iron [30,31,32]. As an important protein involved in iron metabolism, ferritin levels are tightly regulated by iron availability [32,33]. Ferritin expression, i.e., transcription of *FTH1* and *FTL* in the host, is also stimulated by proinflammatory cytokines, e.g., interleukin (IL)-6 and tumor necrosis factor (TNF) alpha (TNFα via the nuclear factor (NF)-κB pathway [31,32,33,34,35]. Therefore, ferritin expression is an indication of iron sequestration away from invading pathogens [28]. This phenomenon is explained by decreasing the available iron that is necessary for pathogen survival and improving the function of antimicrobial immune response [24,29]. Disruptions in nutritional immunity pertaining to iron can result in increased susceptibility to infections as the host may develop iron overload [36]. The alterations in the blood pH caused by iron overload can affect the normal functioning of phagocytic cells, e.g., macrophages or neutrophils thus preventing effective killing of the engulfed fungal pathogens [37,38,39]. This failure in elimination aids in immune evasion and contributes to disease progression [25,26,40,41]. Consequently, iron uptake is a decision-making event for host-pathogen interaction and pathogenesis.

Invading pathogens thrive on freely available iron to proliferate and cause disease, elevated serum iron is a predisposing factor for the development of mucormycosis [28,42]. As such, there remains the possibility of applying iron chelators as adjunctive therapy strategies as exploring this avenue could limit or inhibit fungal growth [11,36,43]. Deferasirox, a commercially available iron chelator, is used as iron overload therapy in transfusion, immunocompromised patients and/or those with elevated serum iron, e.g., diabetic and DKA patients [44,45,46]. Interestingly, preclinical data conducted on DKA murine models of *Rhizopus arrhizus* infection found that deferasirox treatment was as effective as liposomal amphotericin B (LAMB) therapy, while combination therapy of deferasirox-LAMB acted synergistically to improve survival [44,46,47].

Whole genome sequencing of *L. corymbifera* recently revealed putative proteins belonging to the three main mechanisms characterized in fungi that are used to acquire iron in the host [48]. The first involves reductive iron assimilation (RIA) and is a sequential process that reduces iron via the ferric reductase Fre5, the re-oxidation of iron by multicopper ferroxidase (ferroxidase) Fet*3* and finally the translocation across the membrane by high affinity iron permease (permease) Ftr1 [39,49,50,51]. In these pathway, three putative copies of the *FRE5* and *FET3* were identified. Uniquely, *L. corymbifera* was shown to possess four putative copies of the iron permease *FTR1* [48]. The second mechanism is the ability to produce and/or utilize low molecular weight siderophores (iron chelators) while the third employs heme oxygenase to liberate iron from heme [52,53,54,55,56]. As such, these pathways are known as virulence determinants for the fungal pathogens [57,58,59,60,61].

Iron metabolism holds a central role in the development of mucormycosis [62]. In particular, the reductive iron assimilation pathway (RIA) for iron uptake was reported to be relevant during survival in macrophages, and thus during pathogenesis [63]. In *Rhizopus arrhizus*, another prominent mucormycosis causing fungi, it was shown that the single iron permease (rFtr1) and its partner multicopper ferroxidase (rFet3) are the main iron assimilation pathways. The protein complex was also shown to have a major pathogenic role during *in vivo* infection [64]. Although *L. corymbifera* is the second commonly isolated organism causing mucormycosis, little is known about the virulence mechanisms. As promising results from previous studies demonstrating the importance of the RIA pathway in the virulence of mucoralean fungi, the main objective of this work was to identify the components of the reductive pathway. Genome analysis identified the four copies of the genes encoding the permease exhibiting high similarity to the *FTR1* of *S. cerevisiae*, and three copies of the ferroxidase interaction partner with the *FET3* of *S. cerevisiae.* With a focus on the multiple copies of the *FTR1* genes, we sought to study their functional role under iron stress conditions. Here, we have investigated the expression of the genes involved in RIA and have characterized the function of the four putative copies of the *FTR1* under iron stress and during interaction with the host cells [65,66,67]. It was demonstrated that glucose-regulated protein-78 (Grp78/Hsp5a) or heat-shock protein-70 (Hsp70/Hspa8) is abundant on the membrane of macrophages [65,66,67,68]. Most importantly, Hspa8 was shown to be involved in the recognition of *L. corymbifera* spores [67,69]. Therefore, we also sought to deduce the expression pattern of *HSPA8* in macrophages during this interaction. Additionally, we also examined the expression of the iron storage genes in the host, using ferritin (*FTH1*) and in *L. corymbifera* using ferritin (*LcFER* I and II) to gain a brief insight into iron metabolism during the interaction [31].

## 2. Materials and Methods

### 2.1. Strains, Plasmids, and Culture Conditions

Strain of *Lichtheimia corymbifera* JMRC: FSU: 09682 was obtained from the Jena Microbial Resource Collection (JMRC, Jena, listed as WFCC no. 919 at the World Federation for Culture Collections). *L. corymbifera* was routinely maintained on SUP-Medium (10 g/L glucose, 4 g/L KH_2_PO_4_, 0.9 g/L K_2_HPO_4_, 0.25 g/L MgSO_4_ 7H_2_O, 1 g/L NH_4_Cl, and 18 g/L agar) and grown at 37 °C for seven days. Spores were harvested by flooding the plate with 10 mL phosphate buffer saline PBS (1× PBS; 3 M NaCl, 0.5 M KH_2_ PO_4_, 0.27 M KCl, 0.5 M Na_2_ HPO_4_ at pH 7) and gently scrapping the aerial mycelium. Spores were filtered through a 40 µm cell-strainer (Greiner Bio One, 542040, Frickenhausen, Germany)), counted with a hemocytometer and diluted to desired concentration.

All strains used in this study are listed in Table S1. Parent and *S. cerevisiae* strains lacking the iron permease (*FTR1*) were purchased from Dharmacon-Horizon Discovery (https://horizondiscovery.com, accessed on 7 May 2019). The *S. cerevisiae FTR1* null mutants (Δ*FTR1*) were transformed with the plasmid pYES2-*LcFTR1* [70] containing a copy of the putative *L. corymbifera* high affinity iron permease *LcFTR1 I, II* and *IV* (LCor01036, LCor06326, LCor00518, respectively) (Table S2). Native genes were amplified using the gene specific primers *gFTR1* I-IV (Table S3). Gene expression in the plasmid was driven by the inducible TetOn promoter which can induce expression of the putative *LcFTR1* genes by addition of tetracycline in the medium. Previous attempts to express *LcFTR1-I-IV* under the control of *GAL1* promoter failed due to interference with galactose metabolism during iron uptake. The induction of the *GAL1* promoter is inhibited under iron-deficient conditions because the galactose-1-phosphate uridyltransferase (*GAL7*) is a metalloprotein that contains Zn^2+^ and Fe^2+^ [70]. Therefore, new plasmids were constructed by replacing the *GAL1* promoter by a promoter that is inducible by low concentrations of tetracycline (Figure S1). The plasmid was constructed from multiple copies of plasmid which contained *URA3* as a selection marker [71]. The transformants were grown and maintained on Synthetic Complete medium (SC medium: 0.67% (*v/v*) yeast nitrogen base (YNB) without amino acids (Formedium, CYN1102), 2% (*v/v*) glucose plus 0.079% (*v/v*) Complete Supplement Mixtures (CSM) Drop-out: without Uracil (Formedium, DCS0169). Knockout strains were verified by PCR (pYES2_677 and *gFTR1* I-IV) and Sanger sequencing. To induce or suppress the expression of *LcFTR1*, the SC medium was supplemented with or without 25 µg/mL tetracycline, respectively.

### 2.2. RNA Preparation from L. corymbifera

Before each experiment, spores were grown on SC medium with no supplementations compared to supplementation with 20 µM FeCl_3_ and 200 µM extracellular iron chelator bathophenanthrolinedisulfonic acid (BPS) (Sigma-Aldrich, St. Louis, MO, USA, 146617) for 16 h of iron excess and iron depletion, respectively. Total RNA was isolation from ~100 mg mycelia. Briefly, *L. corymbifera* JMRC: FSU: 09682 was cultured overnight in SC medium, mycelia were harvested by filtration using sterile Myra cloth, pressed to remove excess liquid, and immediately frozen with liquid nitrogen. Mycelia were grounded in the presence of liquid nitrogen and RNA was extracted using the RNeasy Plant Mini Kit (Qiagen, Hilden, Germany, 74904). The RNA was treated with RNase-Free DNase set (Qiagen, 79254) and purification repeated using RNeasy Plant Mini Kit. The RNA concentrations were determined using a NanoDrop ND-1000 spectrophotometer (ThermoFisher Scientific, Waltham, MA, United States) and the RNA quality was validated with The QIAxcel RNA QC Kit v2.0 (Qiagen, 929104).

### 2.3. RNA Preparation from Macrophages

#### 2.3.1. Isolation of Monocyte-Derived Macrophages (MDMs)

Buffy coats from healthy human donors were received from the Institute for Transfusion Medicine, Jena University Hospital under the approval of the committee of ethics 4357-03/15 following the Declaration of Helsinki 1975 and 2008. Human peripheral blood mononuclear cells (PBMCs) were isolated according to [72]. Briefly, 5 mL of buffy coat was mixed with 30 mL of PBS and filled up to 50 mL with 15 mL of Biocoll separating solution (Biochrom, Cambridge, United Kingdom, L6155). The solution was centrifuged at 252× *g* for 23 min at room temperature without brake application. The PBMC ring was transferred into new tube and filled up to 50 mL with PBS, then centrifuged for 10 min at room temperature with brake at 160× *g*. The supernatant was discarded and the pellet was dissolved into 1 mL of 1× erythrocyte lysis solution (0.83 g/L NH4Cl, 0.1 g/L of KHCO3, and 0.035 g/L EDTA) for 1.5 min and filled up to 50 mL with PBS. Centrifugation was performed at 112× *g* for 10 min at room temperature. The supernatant was discarded, and the pellet was dissolved into 25 mL of PBS, and then followed by centrifugation step at room temperature for 8 min at 112× *g*. The pellet was resuspended into 1 mL of RPMI-1640 medium and afterwards the number of cells was determined by hemocytometer. The cells were resuspended into RPMI-1640 medium supplemented with recombinant human GM-CSF (Peprotech, Rocky Hill, NJ, USA, 300-03) for seven days for differentiation of macrophages.

#### 2.3.2. RNA Preparation from Mucorales-Infected vs. Uninfected Macrophages

Murine alveolar macrophages (MH-S) ATCC: CRL-2019 (1 × 10^6^ cells per condition) were seeded in 6-well plates and left overnight at 37°C, 5% (*v*/*v*) CO_2_ in RPMI-1640 supplemented with 10% (*v*/*v*) heat inactivated fetal bovine serum. Next, the MH-S were washed twice with the culture media, infected at a multiplicity of infection (MOI) of 5 (5 fungal spores per 1 macrophage) with *L. corymbifera* JMRC: FSU: 09682 and following 3 h co-infection, washed five times with pre-warmed RPMI-1640 medium to remove extracellular spores. At the indicated time point (Control, 3 h, 5 h 8 h, 16 h and 24 h incubation), MH-S cells were scrapped, centrifuged at 400× *g*, and lysed with 450 µL Buffer RLT containing β-mercaptoethanol using the RNeasy Plant Mini Kit (Qiagen, 74904). The following controls were completed in parallel: (1) 1 × 10^7^ *L. corymbifera* spores were grown in RPMI-1640 medium in the absence of macrophages, (2) RNA extraction from uninfected macrophages. Afterwards, isolation of RNA from each sample was performed according to the manufacturer’s instructions.

### 2.4. Quantitative Real-Time Reverse Transcription-PCR (qRT-PCR)

For qRT-PCR reactions, 1 µg of total high-quality RNA was treated with DNase using the RNase-Free DNase set (Qiagen, 79254). RNA was reverse transcribed into cDNA (RevertAid First Strand cDNA Synthesis Kit, Thermo Fisher Scientific, Thermo Fisher, Bremen, 28199, Germany, K1622). The 1 µL diluted cDNA (1:25) used for gene expression analysis by qRT-PCR with EvaGreen Dye (Biotium, 31000, Dresden, 01127, Germany) in Step One Plus (Applied Biosystems QuantStudio 3, ThermoFisher Scientific, Bremen, Germany). The expression rates were done in three independent biological and six technical replicates. The annealing temperature for all primers was 62 °C. The relative standard curve was generated using a pool of cDNAs from all the conditions that were used, which was serially diluted 1:5–1:625. The expression values were calculated/normalized relative to the expression values of the internal control gene ubiquitin conjugating enzyme (UBE, LCor09209.1) (Figure S1) [73]. Additional quality control (QC) and double validation was confirmed using elongation factor 2α (EF2α, LCor01892.1) of *L. corymbifera* (Figure S1) [73,74]. MH-S expression was calculated/normalized relative to the expression values of the internal control gene ubiquitin C enzyme (UC, NM_019639.4) and elongation factor 1α1 (EF1α1, NM_010106) [75]. All primers used for qRT-PCR amplification in single and in combination with macrophages are listed in Tables S4 and S5, respectively.

### 2.5. Iron Titration Assay

SC medium supplemented with 2% (*v*/*v*) Glucose, amino acids and 200 µM iron chelator bathophenanthrolinedisulfonic acid (BPS) (Sigma-Aldrich, 146617), 100 µM, 200 µM or 1 mM FeCl_3_ (Roth, P9742.1) were inoculated with 5 × 10^7^ spores in 100 mL grown at 37 °C, 170 rpm shaking. Samples of 500 µL were taken at 3, 4, 5, 8 and 16 h from each condition, spun down briefly and representative images were captured using a microscope at 400× magnification (Zeiss microscope, Jena, Germany). The number of resting (2–4 µm diameter), swollen (5–8 µm diameter) and germinated spores were enumerated for all time points and conditions to determine an adequate sampling at various time points. The measurement was performed in biological triplicates.

### 2.6. Iron Toxicity Assay

Spores from one SUP agar plate were harvested as previously described. Briefly, 1 × 10^4^ spores in 20 µL were inoculated to the center of a SC medium supplemented with 0.67% (*v*/*v*) YNB, 2% (*v*/*v*) Glucose, 0.079% (*v*/*v*) CSM (Formedium, DCS0169) and 0, 0.1, 0.2, 0.5, 1, 1.5, 2, 3, 4 or 5 mM FeCl_3_. The diameter of mycelial growth was measured daily for seven days. The measurement was performed in biological triplicates.

### 2.7. Iron Assimilation Assay

Strains were cultivated for seven days at 37 °C (*L. corymbifera*) in SD medium (SD: 0.67% (*v*/*v*) YNB, 2% (*v*/*v*) Glucose, 0.079% (*v*/*v*) CSM (Formedium, DCS0169, Norfolk, United Kingdom). Spores were harvested by flooding the plate with 10 mL phosphate buffer saline 1% (*v*/*v*) PBS with 50 µM BPS (Sigma-Aldrich, 146617). 1 × 10^4^ spores/mL were transferred to 200 µL citrate-buffered SD (pH 7.3) with or without 200 µM BPS, 100 µM or 1 mM FeCl3 (stock in 1% (*v*/*v*) HCl), 100 mg/mL horse ferritin (stock in iron-free 5 mM HEPES, 0.15 M NaCl, 4_filtered through 50 kDa molecular weight cut-off columns (Amicon Ultra 0.5 mL, C82301, Darmstadt, Germany), 100 mg/mL transferrin (Calbiochem, 616397 stock in 0.15 M iron-free Na_2_CO_3_, 2× filtered through 50 kDa columns (Amicon Ultra 0.5 mL, C82301), 0.1 mg/mL bovine hemoglobin (Sigma, 9008-02-0, stock in iron-free water (Roth, T143.3)), or 1 mM hemin (Sigma, 16009-13-5, stock in DMSO, Darmstadt, Germany). Growth was recorded 32 h, time point taken at 3 h and 7 h (swollen and germlings, respectively) in biological triplicates by OD_600_ measurement every 30 min (with intermittent 170 rpm shaking) at 37 °C in a Tecan Infinite 200 ELISA reader.

### 2.8. Plasmid Construction and Insert Verification for Heterologous Expression in Yeast

*L. corymbifera* JMRC: FSU: 09682 was cultured overnight in liquid SUP media. The mycelia were harvested by filtration using sterile Myra cloth, pressed to remove the excess of the medium, and immediately frozen with liquid nitrogen. Mycelia were grounded in the presence of liquid nitrogen and RNA was extracted using the RNeasy Plant Mini Kit (Qiagen, 74904). The RNA was treated with RNase-Free DNase set (Qiagen, 79254) and purification repeated using RNeasy Plant Mini Kit (Qiagen, 74904). The RNA concentrations were determined using a NanoDrop ND-1000 spectrophotometer (Thermo Fisher Scientific). RNA was reverse transcribed into cDNA according to manufacturer’s guidelines (RevertAid First Strand cDNA Synthesis Kit, Thermo Fisher Scientific, K1612). Target genes of interest were amplified by polymerase chain reaction using Q5 High-Fidelity 2X Master Mix (New England BioLabs, M0492S/M0492L, Frankfurt am Main, Germany) from cDNA using the following cycling conditions: 98 °C/5 min, then 30 cycles of 95 °C for 10 s, 57 °C for 30 s, 70 °C 2 min and a final elongation at 70 °C for 4 min. Plasmids were linearized using *Eco*RI-HF and *Hind*III-HF (New England Biolabs, R3101 and R3104). Plasmid and the PCR amplified insert with overlapping fragments were gel purified using GeneJET Gel Extraction Kit (Thermo Fisher Scientific, K0691/K0692) and cloned using the In-Fusion^®^ HD cloning kit (Takara, 638920, Göteborg, Sweden) (Table S3). Verification of constructs were confirmed by PCR and sequencing. The generated plasmids were used to transform *S. cerevisiae FTR1* null mutants.

#### Iron Consumption

SD medium: SD: 0.67% (*v*/*v*) YNB, 2% (*v*/*v*) Glucose, 0.079% (*v*/*v*) CSM, (Formedium, DCS0169, Norfolk, United Kingdom) were prepared with 0, 50 µM and 350 µM FeCl_3_ each with and without 25 µg/mL tetracycline. Each well of a 24-well plate was prepared with 1 mL of medium and inoculated with 10 µL (1 × 10^4^ cells) of an overnight culture of all transformant strains, the WT and the Δ*FTR1* mutants *S. cerevisiae*. Following overnight incubation at 30 °C, the cells in each well were suspended and 500 µL of the culture transferred to an Eppendorf tube. The OD_600_ of the remaining 500 µL was measured in all wells. The cultures were centrifuged at 22,000× *g* for 5 min. The assay was performed as described by Tamarit et al. 2006 with the following modifications, volume used 200 µL [76]. Briefly, 20 µL of each supernatant and standard were added to 180 µL of a reaction mixture (3% (*v*/*v*) HNO_3_, 200 mM sodium ascorbate, 3 mM BPS, 1.5 M sodium acetate) in a 96-well plate. As BPS is bright red following iron-chelate complex with an absorption maximum at 535 nm with free Fe^2+^, the sodium ascorbate was added as a reducing agent to allow the detection of Fe^3+^. After addition of the reaction mixture, the plates were incubated for 10 min at RT and the OD of all samples measured at 535 nm with a reference measurement at 680 nm.

### 2.9. Phagocytosis of S. cerevisiae—Expressing FTR Genes by Macrophages

#### 2.9.1. Confrontation Assay

The capability of murine alveolar macrophage cells (MH-S) to phagocytose the *S. cerevisiae*-expressing various *FTR* genes of *L. corymbifera* (*FTR1* copy 1 and copy 2), empty vector (EV), and Δ*FTR1* knock out strain was carried out as previously described by Hassan et al. (2019) with some modifications [69]. Yeast cells were grown in liquid YNB medium supplemented with 2% Glucose, amino acids, 200 µM FeCl_3_ and 200 µg/mL Tetracycline, for 5 days at 30 °C with 170 rpm shaking. The yeast cells were harvested by centrifugation at 2800*× g* for 5 min. The cells were washed twice with phosphate buffer saline solution (1x PBS; 3 M NaCl, 0.5 M KH_2_PO_4_, 0.27 M KCl, 0.5 M Na_2_HPO_4_ at pH 7). The yeast cells were co-incubated with 2 mL of fluorescein isothiocyanate solution, (Sigma Aldrich, 46950, Darmstadt, Germany) (0.1 mg/mL FITC in 0.1 M Na_2_CO_3_) at 30 °C for 30 min with 180 rpm shaking. The yeast cells were washed three times with PBS to remove excessive FITC staining. Cell counts were determined manually using a Neubauer chamber. Finally, the number of yeast cells for each strain was adjusted to a final concentration of 10^6^ per 500 µL of RPMI-1640 medium. 2 × 10^5^ MH-S cells (ATCC CRL-2019™) were diluted in 500 µL of RPMI-1640 medium supplemented with 10% (*v*/*v*) heat-inactivated bovine serum (ATCC-30-2020), 0.05 mM β-mercaptoethanol (Life Technologies, Darmstadt, Germany), 1% (*w*/*v*) gentamycin sulphate (Lonza, 17-518Z, Basel, Switzerland), and (50 mg/ mL^-^) sodium bicarbonate (Lonza, Köln, Germany), and subsequently seeded on glass coverslips (12 mm Ø) in 24-well plates (NUNC). The cells were incubated in a humidified CO_2_ incubator (5% (*v*/*v*) CO_2_) overnight at 37 °C for adherence. The FITC–stained yeast cells were confronted with MH-S cells at multiplicity of infection (MOI) 5 (5 yeast cells: 1 MH-S) and centrifuged at room temperature for 5 min at 100*× g*. The cells were co-incubated for one hour in a humidified CO_2_ incubator (5% (*v*/*v*) CO_2_). The cells were washed three times with ice cold PBS to stop the phagocytosis process and to remove the excess of yeast cells. The cells were co-incubated for ten minutes at room temperature with 500 µL of calcofluor white (CFW; Sigma Aldrich, 18909) (0.5 mg/ mL in PBS). Staining with CFW helps to distinguish the phagocytosed yeast cells from non-phagocytosed ones as CFW cannot penetrate the cell membrane of MH-S cells, therefore phagocytosed yeast cells were stained only with FITC and non-phagocytosed yeast cells were stained with FITC and CFW. The cells were washed twice with ice-cold PBS to remove excess of CFW. Fixation process was performed through co-incubating the cells with 500 µL 3.7% (*v*/*v*) formaldehyde in PBS at room temperature for 15 min.

Microscopy images were acquired by an Axio Observer 7 Spinning Disk Confocal Microscope (ZEISS, Jena, Germany) and processed with ZEN 2.1 Software (ZEISS). At least 30 images were acquired for each strain in each replicate. Three independent biological replicates were performed for each strain.

#### 2.9.2. Automated Image Analysis

The images were analyzed as described previously [77]. Briefly, the outline of MH-S cells was determined by applying a Hessian filter with 3- pixel radii to the transmitted light bright- field (TL-BF) images. The corresponding matrices with the smallest eigen values were utilized as images and they depicted the curved edges of the macrophage cells. The phagocytosed and non-phagocytosed yeast cells were determined based on the fluorescence labeling, as described earlier [77,78] with an additional de-noising filter being included at the beginning of the green signal processing workflow (median filter with 3-pixel radius). Here the green fluorescence channel identified the yeast cells labeled with FITC (all yeast cells were labeled green, i.e., both phagocytosed and non-phagocytosed yeast cells), whereas the blue channel identified only those that were labeled with CFW, corresponding to the non-phagocytosed yeast cells. The phagocytosis measures were calculated via overlapping the regions of interest of segmented MH-S cells with those of green and blue yeast cells as described before [78,79].

### 2.10. In Silico Analysis, Databases and Statistics

The information about gene orthologs, protein structures, and BLAST results were obtained in the respective genome database. BLAST search for the proteins with a predicted multicopper ferroxidases (Fet3) and iron permeases (Ftr1) were performed using the domain sequences for ScFet3 and ScFtr1 as queries. Additionally, the presence of additional conserved protein domains sequences was predicted by SGD-associated prediction tools (SignalP). Analysis was performed using GraphPad Prism software (v7) GraphPad Inc was used for statistics. All data were reported as the mean ± SEM or standard deviation where appropriate and a two-tailed, unpaired Student’s *t*-test was performed if not otherwise indicated. Statistically significant results were marked as ns indicates as non-significant, * *p* < 0.05, ** *p* < 0.01, *** *p* < 0.001.

## 3. Results

### 3.1. Identification of Ferric Reductases in. L. corymbifera

**Figure 1:**
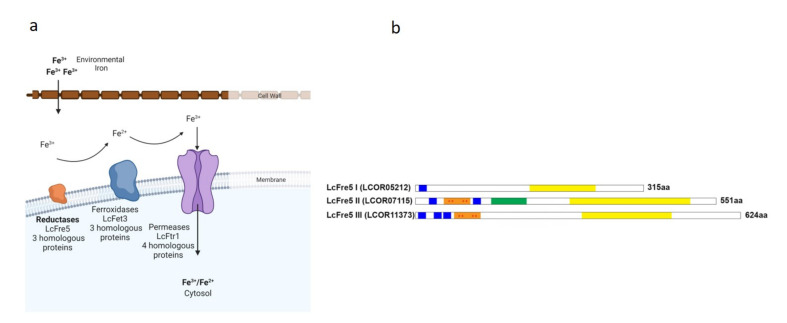

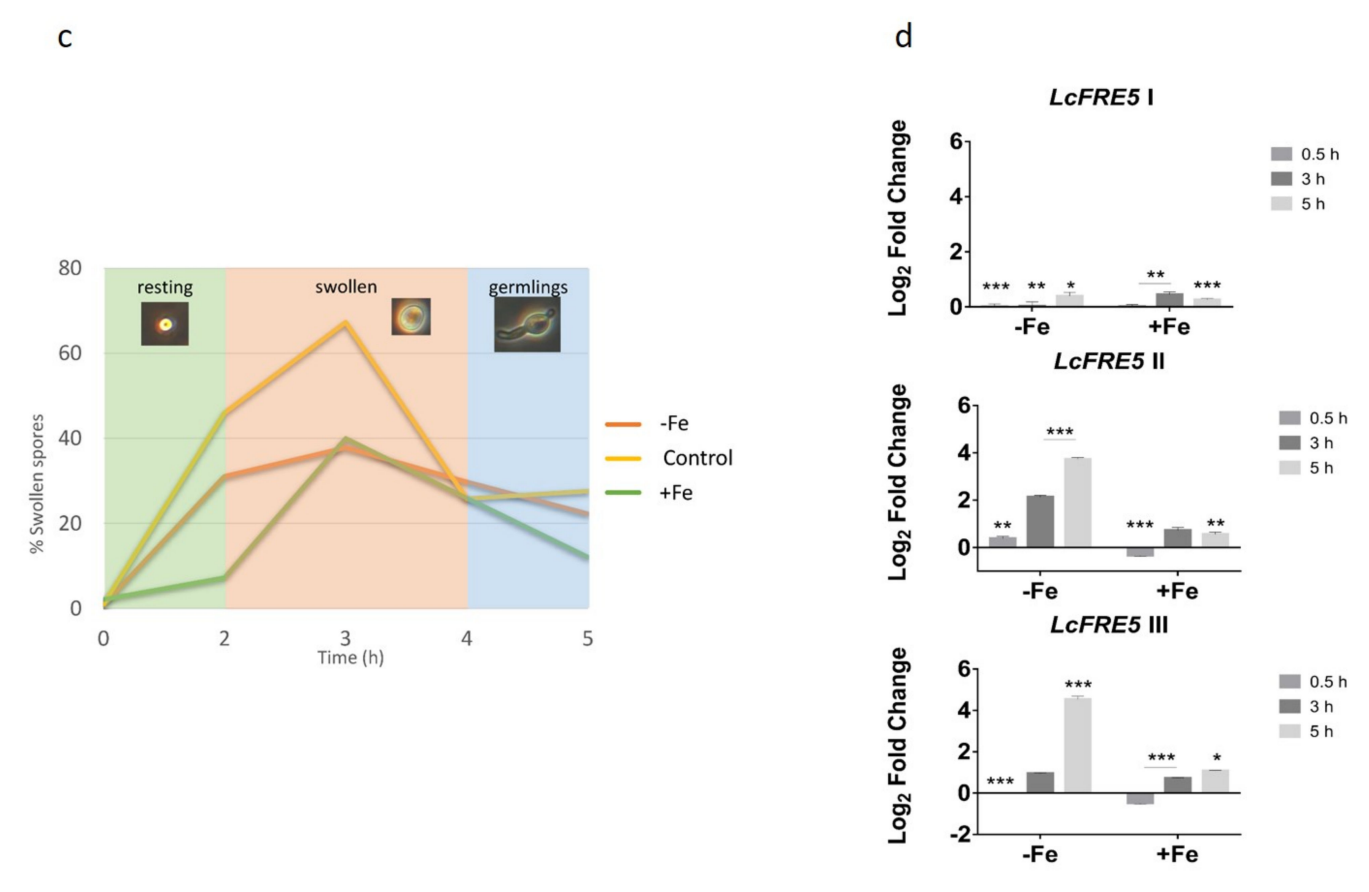
Simplified model of the reductive iron assimilation (RIA) pathway in *L. corymbifera*. (**a**) Putative proteins identified for the RIA in *L. corymbifera*. (**b**) Schematic representation—*L.* *corymbifera* transmembrane ferric reductases present typical domains: ferric reductase domain (orange box); FAD-binding domain (green box); and NAD binding domain (yellow box). Asterisks (red) indicate the conserved histidine residues (4 conserved residues) belonging to the bis-heme motif. Transmembrane region indicated by the blue boxes. The number of amino acids (aa) of each protein is indicated adjacent to the sequences. (**c**) Percentage germination of spores under different iron conditions over time; Control medium supplemented with 0.2 mM FeCl_3_; −Fe (iron depletion) simulated using 0.2 mM BPS; +Fe simulated using 1.0 mM FeCl_3_. (**d**) Early expression profile (log_2_-fold change) relative to the control of the putatively encoding *L. corymbifera* ferric reductase (*LcFRE5* I-III) under iron starvation (0.20 BPS) and iron stress (1.0 mM FeCl_3_). Asterisks indicate statistical significance determined by Student’s *t*-tests comparing treatment and control conditions (* *p* < 0.05; ** *p* < 0.01; *** *p* < 0.001). Data are means and standard error of three independent experiments. Illustrations created with BioRender.com (accessed on 25 January 2021).

The genome of the *ex-type* strain of *L. corymbifera* JMRC: FSU: 09682 was used to identify proteins belonging to the reductive iron assimilation (RIA) pathway (Figure 1a) [48]. An important component of the RIA pathway is the ferric reductases or Fre proteins [80,81]. Three putative copies were found in *L. corymbifera* that exhibit homology to the *S. cerevisiae* ferric reductase (Fre5) (Figure 1a). These genes were identified as *LcFRE5* I-III (LCor05212.1; LCor07115.1 and LCor11373.1, respectively) [48]. Amino acid alignment showed only two copies of the protein (LcFre5 II and III) contain the ferric reductase domains (Figure 1b) and Figure S2 (Figure S3). LcFre5-III contains a C-terminal cytoplasmic FAD-binding and while all three copies possess NAD-binding domains [23,24]. Moreover, amino acid sequences belonging to the putative ferric reductases LcFre5 homologs were used to align the amino acid sequences along with ferric reductases from *S cerevisiae, Candida albicans*, *Aspergillus fumigatus* and *Cryptococcus neoformans* (Figure S3).

Deviation from norm-iron conditions appears to hamper spore swelling in *L. corymbifera* as shown for iron-limited and iron-stress conditions (Figure 1c). This prompted us to investigate the expression of the genes involved in the RIA pathway. Expression analysis of the putative *LcFRE5* copies (I-III) indicate that all three copies show a moderate change in expression under iron-rich conditions but are differentially expressed in iron-limited environments (Figure 1d). Whilst *LcFRE5*-I does not appear to play any leading role under iron starvation while both *LcFRE5*-II and -III are strongly upregulated under iron-depleted conditions (Figure 1d).

### 3.2. Identification and Expression of Iron Permease (FTR1)-Iron Ferroxidase (FET3) Syntenies in L. corymbifera

**Figure 2:**
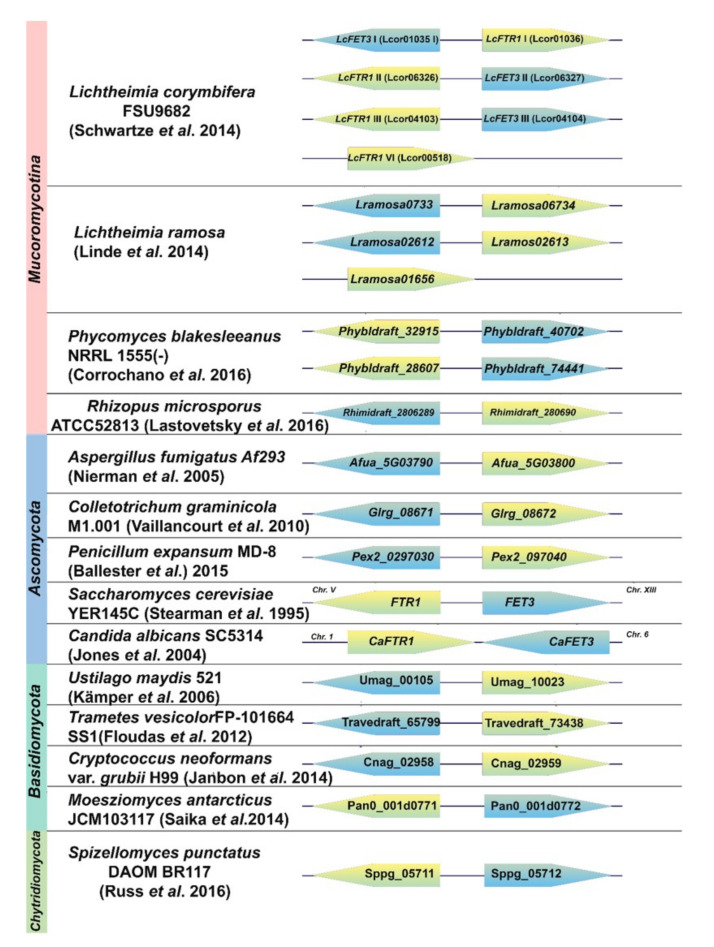
Synteny of Ftr1 (green) and Fet3 (blue) orthologue genes in different fungal division; all genes included were annotated as *FTR1/FET3* or found to closely match known genes in BLAST analysis; labels represent the locus tag in the annotated genome; arrows indicate coding orientation; arrow length not representative of protein sizes, all data were obtained from the NCBI database [48,49,84,85,86,87,88,89,90,91,92,93].

**Figure 3:**
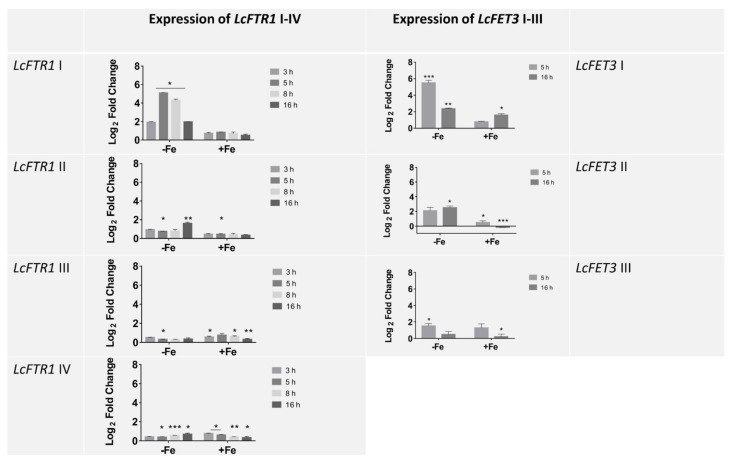
Expression profile represented as log_2_-fold change relative to the control condition of the putatively encoding *L. corymbifera* iron permeases (*LcFTR1* I-IV) and the partner ferroxidase *LcFET3* (*LcFET3* I-II). Left panel: gene expression of the *LcFTR1* I-IV under iron depleted, represented as −Fe (0.20 mM BPS) and iron stress simulated using (1.0 mM FeCl_3_. Expression represented as log_2_-fold change relative to each control condition. Right panel: gene expression of the *LcFFET3* III for 5 h and 16 h under: iron depleted (0.20 mM BPS) and iron excess (1.0 mM FeCl_3_) conditions; data are means and standard error of three independent experiments. Asterisks indicates statistical significance determined by Student’s *t*-tests comparing treatment and control conditions at each time point (* *p* < 0.05; ** *p* < 0.01; *** *p* < 0.001). Data are means and standard error of three independent experiments.

The genome of *L. corymbifera* contains four copies of the iron permease genes, i.e., *LcFTR1*-I-IV (Table S2, Figure 2). Examining the gene organization, the fourth copy lacks the coupled ferroxidase *LcFET3* (Figure 2). This synteny observed in the genome organization of *L. corymbifera* follows that organization seen in pathogenic and non-pathogenic fungi (Figure 2) [82,83]. The multicopper ferroxidase (*FET3*, syn. ferroxidase) is coupled with the iron permease (*FTR1*) in a *FTR1*-*FET3* synteny which is shown by co-regulation of *LcFTR1* and *LcFET3* as observed by co-expression of their corresponding copies *LcFTR1-I-III* with *LcFET3*-I-III, respectively (Figure 3). The expression of the high-affinity iron permeases is known to be induced under iron-depleted conditions and repressed in iron-rich environments [63]. It would appear that *L. corymbifera* possesses a dominant copy of *LcFTR1 I* (LCor01036.1) that is highly expressed under iron starvation (Figure 3). There is a peak in expression at 5 h post-exposure to iron depletion marking germination and early hyphal development followed by a gradual decrease in the transcript levels. Interestingly, the second copy *LcFTR1* II (LCor06326.1) is constitutively expressed during 3–8 h, but moderately upregulated at 16 h marking the later hyphal development stage. This shift in expression pattern suggests a possible developmental specialization by which *LcFTR1* I is responsible for iron uptake during the earlier hyphal development, which is supported by *LcFTR1* II during the later hyphal development. A detailed statistical analysis on the significance among the expression of the *LcFTR1* gene copies is shown in Tables S6 and S7. The remaining two copies of the permeases *LcFTR1* III (LCor04103.1) and *LcFTR1* IV (LCor00518.1) appear to be constitutively expressed during all developmental stages during iron depletion. Under iron supplementation, no differential expression was observed for all four copies of *LcFTR1*. Overall, there is a pattern of iron regulation of the permease transcription abundance in *L. corymbifera*.

Three putative copies of the ferroxidases were identified in the genome of *L. corymbifera* JMRC: FSU: 09682 (Table S2). To investigate the three copies of the ferroxidases, the amino acid sequences were analyzed using the Laccase Engineering Database (LccED: https://lcced.biocatnet.de/, accessed on 30 December 2020). Analysis revealed that *L. corymbifera* sequences were classified in family E, corresponding to fungal ferroxidases. The ferroxidases were also classified as basidiomycetous laccases (family A), ascomycetous laccases (family B), and fungal pigment ferroxidases (family D). The putative ferroxidase sequences were aligned with the *S. cerevisiae* ferroxidase (*ScFET3*) (Figure S4) to identify potential carboxylate side chains that are known to support the ferrous oxidase activity in fungal ferroxidases, as well as copper (Cu) binding residues [94]. The alignment revealed that two copies, LcFet3 I and II (LCor01035 and LCor06327, respectively) correctly display the four Cu-binding motifs required to support the ferroxidase activity typical in the RIA pathway. Ferroxidase activity in fungal multicopper ferroxidases (Fet3) requires two acidic residues, E185 and D407; LcFet*3* I and II have both residues while LcFet*3* III (LCor04104) lack E185 (Figure S4). As such, the former two are likely ferroxidases and the latter copy may have lost its ferrous oxidase activity. Expression analysis showed that both *LcFET3* I and II were upregulated under iron starvation at 5 and 16 h (Figure 3). Matching with the partner *LcFTR1* I, *LcFET3* I showed the highest expression at 5 h iron starvation and was downregulated in iron-rich conditions (Figure 3). The expression levels of *LcFET3* III remained moderately unchanged under both conditions that strongly indicating *LcFET3* III may not be a functional ferroxidase (Figure S4). Collectively, isolation and characterization of these proteins are necessary to determine their possible ferroxidase activity.

### 3.3. Iron Resistance of L. corymbifera

**Figure 4:**
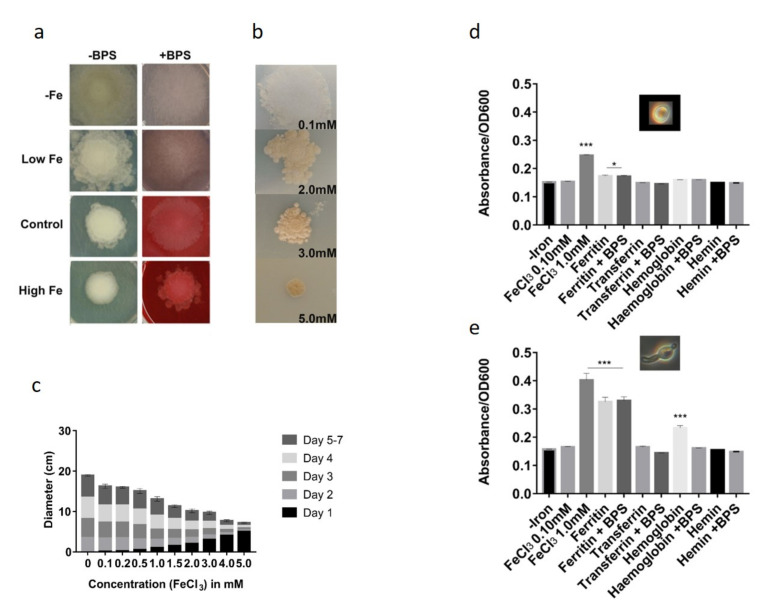
*L. corymbifera* growth under iron stress. (**a**) *L. corymbifera* cultures were harvested after seven days of growth on SC medium supplemented with 0.10 mM FeCl_3_, washed and 10^4^ spores were spotted on SC medium supplemented or not with 0.2 mM BPS and 0.1 mM, 0.2 mM and 1.0 mM FeCl_3_. (**b**) Phenotypic comparison of the *L. corymbifera* growth under iron stress conditions. Growth on SC medium supplemented with 0.1, 2.0, 3.0, and 5.0 mM FeCl_3_. (**c**) Mycelial growth on increasing concentrations of iron for 7 days. Diameter measurements were taken from triplicate plates. Error bars indicate the standard error at day 7. (**d**) Germination of *L.* *corymbifera* spores in different iron sources: spores were inoculated into liquid SC medium supplemented or not with 0.2 mM BPS and ferritin, transferrin, hemoglobin and hemin (100 µg/ mL) independently. * *p* < 0.05, *** *p* < 0.001.

As *L. corymbifera* may possess a main Ftr1 permease that utilized during iron-depleted condition, spores were cultivated for seven days in the presence of increasing concentration of inorganic iron to determine the minimum inhibitory concentration of iron for *L. corymbifera* (Figure 4). At higher concentrations of iron, i.e., 3–4 mM FeCl_3_, a yeast-like morphology was observed with surrounding satellite colonies that lack the typical grey/white aerial hyphae as seen at 0.1 mM concentration of FeCl_3_ (or normal conditions) indicating tolerance of *L. corymbifera* to higher iron concentration (Figure 4a,b). However, fewer spores were harvested from 3 mM and 5 mM FeCl_3_ supplemented plates. Additionally, there were visible malformations in the sporangia and hyphae of the *L. corymbifera* grown at higher concentrations (3–5 mM). To quantify the hyphal growth, the colony diameter was measured daily for seven days (Figure 4c). The largest diameter was observed on plates that were not supplemented with FeCl_3_, thus indicating that iron storage of *L. corymbifera* is robust. Growth was slower at higher concentrations (1.0–4.0 mM) indicating adverse effect of iron excess on the growth of *L. corymbifera*. Significant drops in growth were observed between the concentrations of 3 and 5 mM FeCl_3_ (Figure 4c). Assimilation of different iron sources was done to determine the best growth-promoting iron source (Figure 4d,e). Whereas iron-excess given by 1 mM FeCl_3_ seems to inhibit spore germination at swollen spore condition as indicated by higher turbidity at OD_600_, ferritin and hemoglobin appeared to benefit filamentous growth during germling condition (Figure 4d). This indicates that *L. corymbifera* may be able to utilize these host iron sources. Interestingly, ferritin coupled with BPS cultures grew similarly to ferritin only suggesting that the *L. corymbifera* is equip at scavenging iron under stress.

### 3.4. Expression of FTH1 and LcFER as Iron Storage Markers over Different Time Points

**Figure 5:**
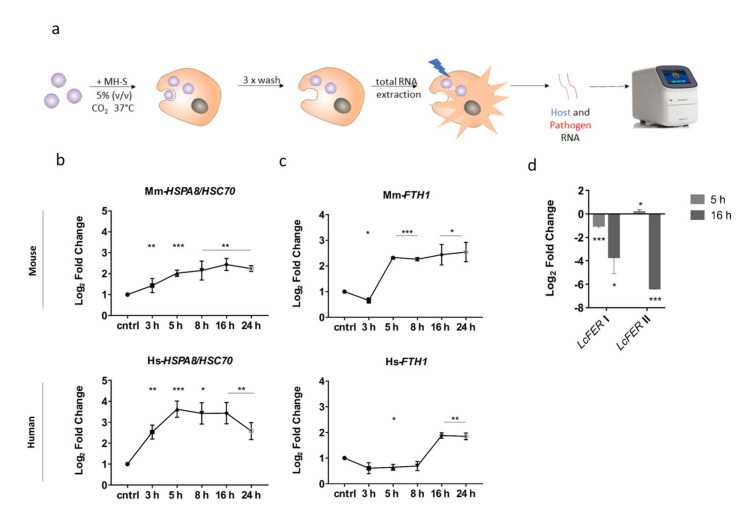
Expression profile of host protein Hspa8 (*HSPA8*), iron storage (*FTH1*) in macrophages and *L. corymbifera* during interaction for 24 h. (**a**) Schematic representation of workflow used for host pathogen interaction and gene expression validation. (**b**) Expression profile of the murine alveolar macrophage (MH-S) protein Hspa8 (*HSPA8*) and human Hspa8 protein (*HSPA8*) during interaction with *L. corymbifera* spores. (**c**) Expression pattern for host iron storage genes (*FTH1*) in murine alveolar macrophages (MH-S) and human macrophages. **(d**) Changes in gene expression for the putative copies for iron storage Ferritin (*FER* I and *FER* II) of *L. corymbifera*. Data are means and standard error of three independent experiments. Asterisks indicates statistical significance determined by Student’s t-tests comparing treatment and control conditions at each time point (* *p* < 0.05; ** *p* < 0.01; *** *p* < 0.001).

Confrontation of *L. corymbifera* spores with macrophages (MH-S cells) showed an upregulation of the *HSPA8* gene encoding the 71 kDa heat-shock protein Hspa8/Hsc70 in MH-S cells which was previously shown as an important factor for recognition and the phagocytosis of *L. corymbifera* [67]. The measurement of *HSPA8*-expression was used as marker for successful interaction between *L. corymbifera* spores with MH-S cells (Figure 5a). Examining the expression pattern showed that *HSPA8* higher transcript abundance in the human macrophages in comparison to the murine macrophages (MH-S). In both cases, these results strongly indicate that exposure to the spores increases Hspa8 protein trafficking to the cell surface thus increasing phagocytosis of invading *L. corymbifera* spores. Additionally, transcript levels in both macrophages were higher during the early stages of interaction (3 h–16 h). However, there was a marked reduction at 16 h interaction. The expression of *FTH1* (encodes the heavy subunit of ferritin, the major intracellular iron storage protein in pro- and eukaryotes) was measured during the macrophages interaction to assess the internal iron storage during phagocytosis (Figure 5c). Host iron storage (Figure 5c) also exhibited a difference in the expression level of *FTH1* between murine (MH-S) and human macrophages. In the murine macrophages, there was a slight decline followed by a sharp increase in transcript levels between 3 h–5 h. However, this pattern was not reflected in the human macrophages as the levels remains relatively constant between 3 h and 8 h. This was followed by a marked increase after 8 h interaction in both murine and human macrophages. This pattern suggests that there was an increase production of ferritin which would effectively chelate intracellular iron away from the phagocytosed spores. An interesting pattern was observed at 24 h, where there was a moderate increase in expression of *FTH1* and a reduction in *HSPA8* transcripts in the human macrophages (Figure 5b,c) [95]. The internal iron storage marker ferritin was also used to elucidate the internal iron store on the pathogen side. In *L. corymbifera*, expression of *LcFER* I and II were both strongly downregulated at 5–16 h. This is most apparent at the 16 h time point, where both are strongly downregulated. This expression pattern indicates that iron is no longer being stored as the mucoralean ferritin-like protein is not produced (Figure 5d). This pattern could be an indication of iron mobilization for spore swelling and use against host antifungal defenses, e.g., reactive oxygen species (ROS).

### 3.5. Similarities of LcFTR1 to Most Causative Pathogenic Fungi

**Figure 6:**
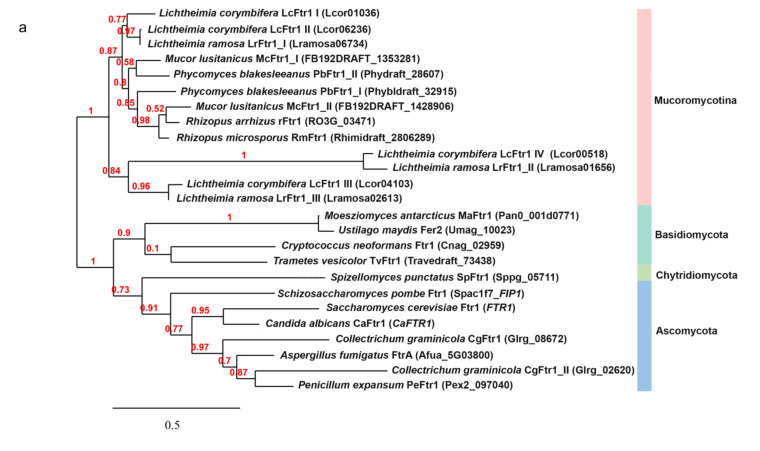

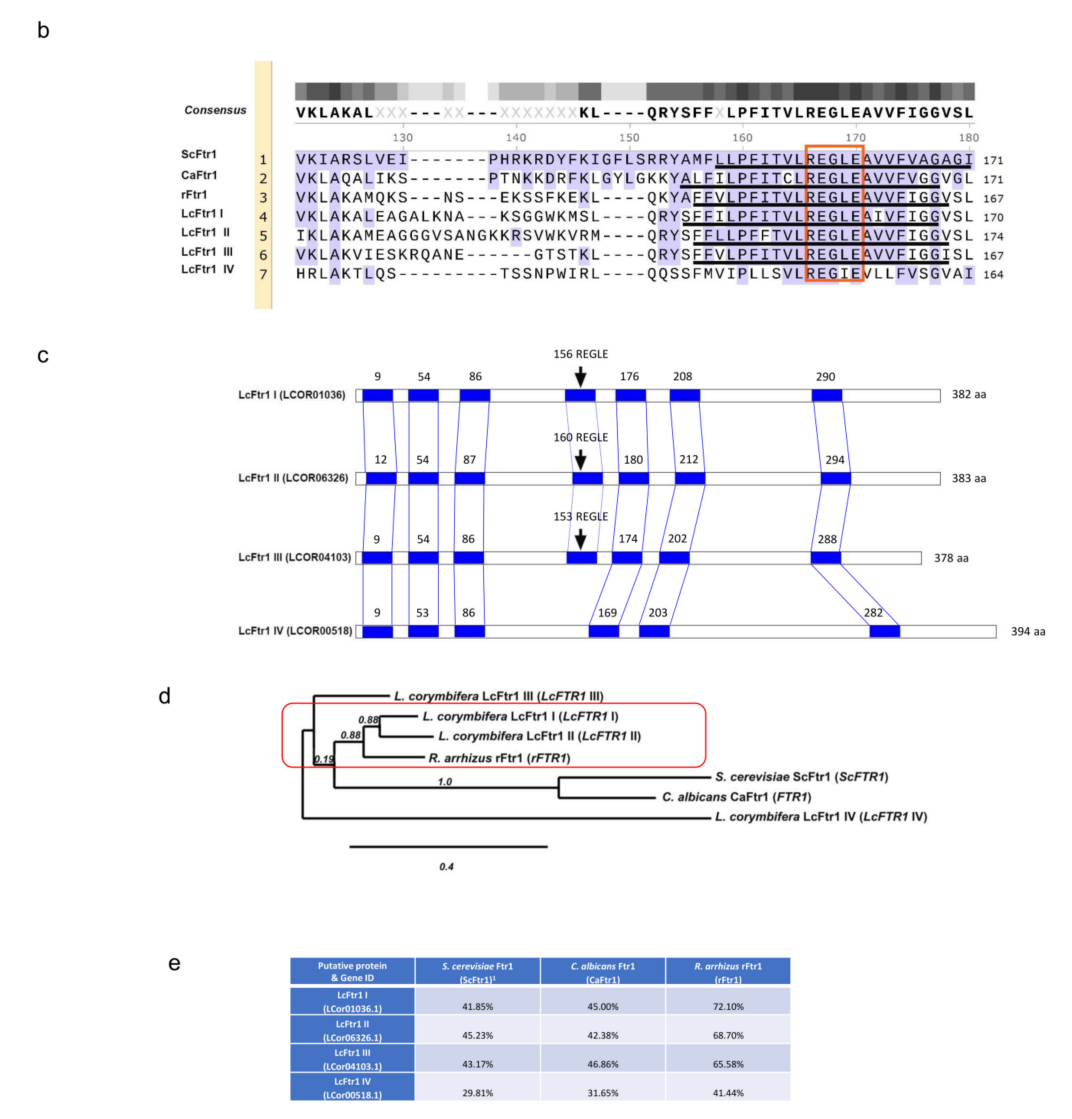
Phylogenetic analysis of the iron permeases (Ftr1) from different fungi. (**a**) Phylogenetic alignment of was completed using the amino acid sequences from different fungi were obtained from the respective genome database or NCBI and used for analysis. The alignment was completed using the maximum likelihood method implemented in the PhyML program (v3.1/3.0 aLRT). The WAG substitution model was selected assuming an estimated proportion of invariant sites (of 0.233) and 4 gamma-distributed rate categories to account for rate heterogeneity across sites. The gamma shape parameter was estimated directly from the data (gamma = 1.620). Reliability for internal branch was assessed using the aLRT test (SH-Like) [102,103,104,105,106,107,108,109]. (**b**) Amino acid sequence alignment indicating the REGLE motif (red box) embedded in the transmembrane domain (black lines) [25]. (**c**) Schematic representation of the predicted transmembrane domains (TM) of the four putative LcFTR1 copies. LcFtr1 amino acid (aa) are illustrated as the rectangle box; predicted TMs are indicating by the blue bars (numbers are the LcFtr1 amino acid numbers referring to the first amino acid of each transmembrane domain). The location of the REGLE motifs is indicated by the arrows (numbers denote the first amino acid of the motif). The number of amino acids (aa) of each protein is indicated adjacent to the sequences. (**d**) Amino acid alignment of the *R. arrhizus* permease (*rFTR1*), *S. cerevisiae* permease (*ScFTR1*) and *C. albicans* permease (*CaFTR1*) with the putatively encoding *L. corymbifera* iron permeases (*LcFTR1* I-IV). Abbreviations: *LcFTR1*: *L. corymbifera; L.ramosa: L. ramosa; Mlusitanicus_FB192DRATF: M. circinelloides f. lusitanicus; Phybldraft: Phycomyces blakesleeanus; RO3G; R. arrihizus (R. delemar); Rhidraft: R. microspores; Pan0: Moesziomyces antarcticus; Umag: Ustilago maydis; Cnag: C. neoformans; Traveldraft: Trametes vesicolor; Sppg: Spizellomyces punctatus; ScFTR1: S. cerevisiae; SCPAC1F7_FIP1: Schizosaccharomyces pombe; CaFTR1: C. albicans; Glrg_ Colletotrichum graminocola; AfuA: Aspergillus fumigatus; Pex2: Penicillum expansum*. (**e**) Identities (in percentage) among putative LcFtr1 proteins in *L. corymbifera*.

Global alignment of the amino acid sequences deduced from the four copies of the *LcFTR1* genes were aligned with the iron permease protein sequences belonging to other prominent fungi (pathogenic and non-pathogenic) (Figure 6a). This analysis showed that *LcFTR1* I and II cluster with one of the *Mucor circinelloides* f. *lusitanicus* permease copies. Here, we see the LcFtr III is clustered separately and close to the third copy of the *L. ramosa* permease (LrFtr1 III), a similar pattern is seen for the permease LcFtr1 IV. The amino acid alignment of *L. corymbifera* LcFtr1 copies with *S. cerevisiae* ScFtr1, *C. albicans* CaFtr1, and *R. arrhizus* rFtr1 highlights the REGLE motif embedded in the transmembrane domain (Figure 6b and Figure S5). The REGLE motif is a conserved sequence that facilitates iron-binding. The permeases belonging to *S. cerevisiae*, *C. albicans*, *R. arrhizus* all possess this conserved REGLE motif (Figure 6b) [71,96]. Interestingly, only three copies of the *L. corymbifera* permeases, i.e., *LcFTR1* I-III contain the REGLE motif while *LcFTR1* IV lacks this sequence. *R. arrhizus* rFtr1 and three copies of LcFtr1(I-III) seven predicted transmembrane domains while *LcFTR1* IV possess six and no REGLE motif (Figure 6c). Multiple regions of the LcFtr1 putative protein sequences are homologous to *S. cerevisiae* and *C. albicans* Ftr1 transmembrane domains (Figure S5) [49,97,98]. Comparison of the putative LcFtr1 (I-IV) with those of the three other fungi showed varying degrees of identities as shown in the cladogram (Figure 6d,e). These results indicate that the LcFtr1 I and LcFtr1 II proteins are paralogs originating from a gene duplication and are most closely related in *L. corymbifera*, and thus orthologous to the previously characterized rFtr1 in *R. arrhizus* (Figure 6d) [64,85,99,100,101]. Whereas LcFtr1 IV exhibit the least sequence similarity to *S. cerevisiae, C. albicans* and *R. arrhizus* indicating that this copy may have evolve towards neo-functionalization. LcFtr1 III appears to originate from another gene duplication of the *LcFTR1* gene which is more ancestral.

### 3.6. LcFTR1 Restores the Ability of the S. cerevisiae Ftr1 Null Mutants and Has an Effect on Phagocytosis by Macrophages

**Figure 7:**
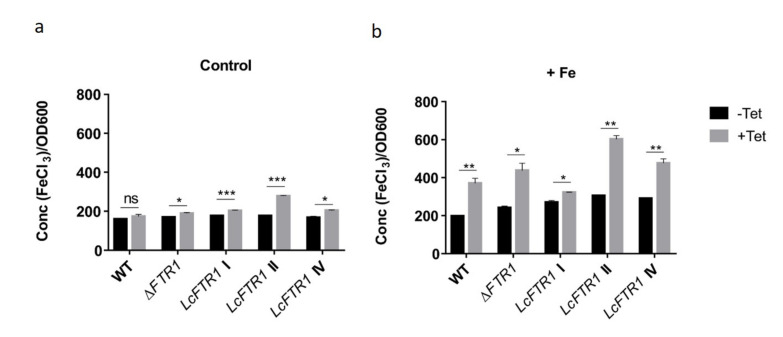

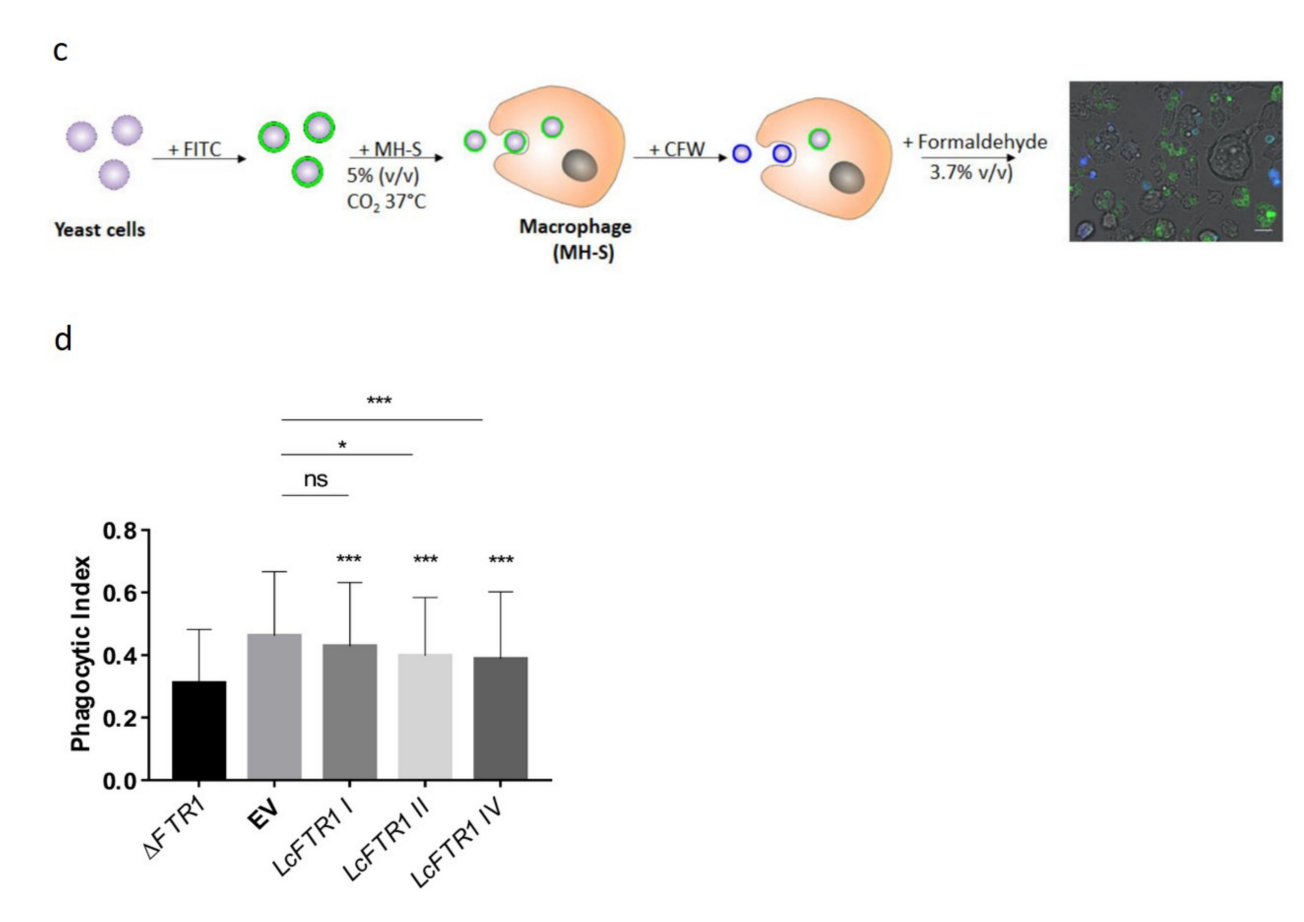
Restoration of iron-dependent growth defect in *S. cerevisiae ΔFTR1* mutants by the three copies of the *LcFTR1* genes. Δ*FTR1 S. cerevisiae* strains were transformed with a vector expressing *LcFTR1* I, II and IV under the control of Tet or vector alone and assessed for iron consumption. (**a**) Control conditions with 50 µM FeCl_3_; (**b**) high iron using 350 µM FeCl_3_. Error bars indicate standard deviation of the triplicate measurements; significant differences between the induced and uninduced samples determined by unpaired Student’s two-tailed t-test are indicated by * (α = 0.002) or ** (α = 0.001). (**c**) Schematic showing the workflow used to for interaction of murine alveolar macrophages (MH-S) with heterologous yeast strains and data output. (**d**) Interaction of murine alveolar macrophages (MH-S) with iron-dependent growth defect of *S. cerevisiae FTR1* null mutants complemented with the 3 copies of *L. corymbifera LcFTR1* genes. *S. cerevisiae FTR1* null mutants overexpressing *FTR1* genes (I, II and IV) were phagocytosed by MH-S. The statistical test was performed based on comparison of over-expressing FTR1 gene of *L. corymbifera* to *S. cerevisiae* expressing empty vector (EV) for vector control without insert. Student’s *t*-test was used to determine the significant difference, as ns denotes non-significant difference, * means *p* < 0.05, ** means *p* < 0.01 and *** refers to *p* < 0.001.

To determine whether *LcFTR1* is functionally equivalent to the characterized *S. cerevisiae FTR1*, we tested whether *LcFTR1* can rescue the iron-dependent growth defect of a *FTR1* null mutant *S. cerevisiae*. *S. cerevisiae FTR1* null mutants were transformed with the pYES2Tet plasmid containing *LcFTR1* copies (*FTR1 I* and *FTR1 II*) under the control of tetracycline (Tet) inducible promoter (Figure S2). *S. cerevisiae FTR1* null mutants were able to grow in SC medium containing Tet and supplemented with 50 µM or 350 µM FeCl_3_ as the low-affinity permeases remain intact (Figure 7a,b). All *S. cerevisiae* transformants expression *LcFTR1* copies grew in the control conditions (50 µM FeCl_3_) with the marked difference in LcFtr1 II (Figure 7a). Iron concentrations relative to OD_600_ of the cultures appear higher for cells expressing *LcFTR1* copies. Interestingly, LcFtr1 II and IV have a noticeable increase in the iron concentrations when compared to the wild-type *S. cerevisiae* in iron-rich conditions (Figure 7b). Again, the higher growth of the *S. cerevisiae FTR1* null mutants in 350 µM FeCl_3_ of iron may be due to intact low affinity uptake systems and/or resistance to higher iron concentrations.

Interaction of the complemented *S. cerevisiae* null mutants (Δ*FTR1*) was accomplished with murine alveolar macrophages (MH-S) in order to mimic a host response scenario (Figure 7c). Confrontation of the yeast transformants with macrophages revealed decreased phagocytosis of *LcFTR1* II and IV overexpressing yeast cells compared to the vector control of yeast cells, whereas the phagocytosis of *LcFTR1* I overexpressing yeast cells remained unaffected (Figure 7d). This indicates a neutral role of *LcFTR1* I, but a suppressive role of *LcFTR1* II and IV in recognition by macrophages.

## 4. Discussion

Whole genome sequencing of *L. corymbifera* JMRC: FSU: 09682 revealed putative components of the three main mechanisms for iron uptake identified in fungi [48,110,111,112]. These pathways include: (1) the reductive iron assimilation (RIA) which involves the reduction of ferric iron followed by oxidation carried out by the multicopper ferroxidase coupled and subsequent transport into the cell by the ferric iron permease [113]; (2) a siderophore permease pathway that facilitates the transport of xenosiderophores (siderophores produced by other fungi and bacteria) [114]; and (3) a heme oxygenase pathway that facilitates iron harvesting from hemin and haemoglobin [48,63,115]. RIA was first characterized in the model organism *S. cerevisiae*, and it was demonstrated that iron transport involves an initial reduction step facilitated by dedicated ferric reductases (Fre1 and Fre2). The reduced iron is then re-oxidized by the multicopper ferroxidase (Fet3) and transported by the partner protein, iron permease (Ftr1) [61,116,117]. In murine models of *C. albicans* infection, it was shown that *FTR1* null mutants had reduced virulence in comparison to wild-type [97,99]. *C. albicans* mutants lacking the ferroxidase showed a similar reduced virulence profile to the null *FTR1* strains in mouse models of systemic candidiasis [110]. In mucoralean fungi, the heterologous expression of the single copy of *R. arrhizus FTR1* (r*FTR1*) in *S. cerevisiae*, was able to restore growth in null mutants [71]. Similarly, heterologous expression of the three most prominent copies of *LcFTR1* (I, II and IV) were also able to restore growth in *S. cerevisiae FTR1* null mutants. Our results also show that *L. corymbifera* Ftr1 I and II may plays a role in iron uptake as *LcFTR1* I increase in expression suggests it may be responsible for initial uptake between 1–4 h. Later, *LcFTR1* II is expressed, and the protein is recruited after 5 h. Most recently, it was shown that both the ferroxidase (rFet3) and permease (rFtr1) are key virulence determinants in murine models as both genes were highly upregulated [64,71]. *Aspergillus fumigatus* possess the three components of the RIA pathway, i.e., iron permease (FtrA), ferroxidase (FetC) and ferric reductase (FreB) [118]. Expression of these genes *FTRA*, *FETC* and *FREB*, are also induced during iron starvation [119,120,121,122,123]. In contrast to *C. albicans* and *R. arrhizus*, complete knockout of *FTRA* does not attenuate virulence of *A. fumigatus* [119,120]. The less pathogenic *A. nidulans* lacks homologs of *S. cerevisiae FTR1* and *FET3* [118,119]. Our results indicate that two of the three copies of the putative ferroxidases in *L. corymbifera* may be functional i.e., *LcFET3* I and II, as these two copies possess the two amino acid residues that are necessary for the functional activity. Recently, the ferroxidases belonging to the *Mucor circinelloides* were characterized and demonstrated that three copies, i.e., *FETA*, *FETB* and *FETC* have functional specialization and were differentially expressed in the yeast and hyphal forms [124]. This strongly indicates that *LcFTR1* I and II may also function as a virulence determinant as their expression is more pronounced in iron-depleted environments.

We focused on characterizing the prominent iron permease of *L. corymbifera* to determine functionality and to further evaluate its role in the growth under iron limited conditions.

In this study, the results strongly indicate that LcFtr1 I may be the main high-affinity iron permease involved in the iron acquisition during initial exposure to iron depleted conditions. However, the second copy LcFtr1 II may be expressed at later developmental stages during iron depleted conditions. Interestingly, this developmental specialization of proteins involved in the reductive pathway has been shown in *M. circinelloides* [124]. Two copies of *LcFTR1* I and *LcFTR1* II showed a considerable homology to *rFTR1*, *CaFTR1,* and *ScFTR1*. The first copy of the iron permease *LcFTR1* I is highly expressed in iron-depleted conditions and suppressed in high iron conditions which strongly indicates that this copy is a high-affinity iron permease [49,64,97,125]. However, during the interaction with macrophages as there was moderately higher phagocytosis of the yeast cells expressing *LcFtr1* II while LcFtr1 IV was the least phagocytosed. On the other hand, interaction of these complemented strains with murine macrophages showed that *LcFTR1* I did not interfere phagocytosis like readily demonstrated by *LcFTR1* II, *LcFTR1* IV and the *FTR1*-deficient mutant of *S. cerevisiae* indicating that *LcFTR1* I may be relevant for immune evasion facilitating intracellular survival and *LcFTR1* II, *LcFTR1* IV may be relevant for recognition of *L. corymbifera* by macrophages. Interestingly, *LcFTR1* I and *LcFTR1* II showed considerable homology to the *rFTR1*. The expression of *LcFTR1* I and *LcFTR1* II successfully restored growth *FTR1* null *S. cerevisiae* mutants in iron supplemented medium.

The interaction of the iron permease Ftr1 with the ferroxidase Fet3 is essential for the correct functioning of the reductive pathway [49,112,113]. Our results indicate that the *LcFTR1* II can interact with the *S. cerevisiae FET3* as it complemented the growth defect in this pathway. On the contrary, the heterologous expression of the *Schizosaccharomyces pombe* ferroxidase *FIO1* in *S. cerevisiae FET3* null mutants could not restore the functioning reductive pathway thus indicating that *S. pombe FIO1* and *ScFTR1* cannot interact [96].

The host cell protein Hspa8 (Grp78) exhibited a high abundance on the cell membrane of macrophages when challenged with *L. corymbifera* spores [67]. As such, we sought to investigate the expression of the corresponding genes *HSPA8*/*HSC70* during interaction with host cells. The increase in expression of this gene over time strongly indicates protein production and potential increase presentation at the macrophage cell surface. These results may further support the previous findings showing increased protein abundance on the macrophage cell surface [67]. Examination of both host iron storage genes, i.e., *FTH1* during interaction showed increased expression over time. This also strongly suggests that there was ferritin production and subsequent sequestration of iron away for the phagocytosed spores which should inhibit intracellular growth [31,32,126]. This pattern is recognized and is a normal response to invading pathogens in healthy phagocytic cells [63,66]. In other fungi iron storage is facilitated by either vacuolar or siderophore-mediated storage [127,128,129]. *S. cerevisiae* utilizes vacuolar storage while *A. fumigatus* employs ferrichrome siderophores as an internal iron storage molecule [120]. However, hydroxyferricrocin is used as the iron storage molecule in conidia while ferricrocin is used in the hyphal stage of growth thus iron storage in *A. fumigatus* has developmental specificity [120,127]. In the Mucoromycota, ferritin-like containing proteins have been identified as potential intracellular iron storage molecules [130]. In *L. corymbifera*, the two putative iron storage genes were identified, *FER* I and *FER* II [48]. Expression analysis showed that both copies were strongly down regulated during phagocytosis. This strong down regulation indicates that the iron storage protein is not being produced and iron storage may be inhibited [130]. *L. corymbifera* mobilization of iron stores from the ferritin pathway could lead to ineffective killing of spores via resistance to oxidative stress. This cascade may allow germination, triggering apoptosis followed by growth and host tissue invasion particularly as typical of mucormycosis [67,114,131].

**Figure 8:**
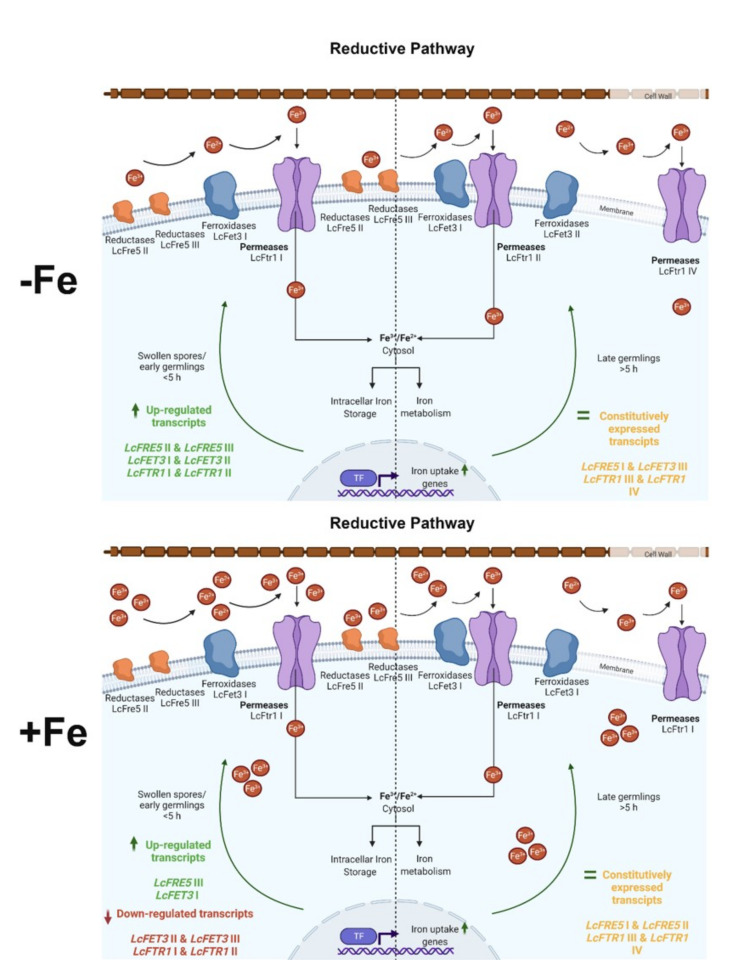
Schematic representation of *L. corymbifera* reductive iron assimilation (RIA): involving the ferric reductases, the multicopper oxidases and the ferric iron permeases. Differentially expressed genes belonging to the RIA during iron depletion (upper panel) and high iron (lower panel). Upregulated transcripts are indicated in bold colors; green represents upregulations, i.e., high expression of *LcFTR1/LcFET3* transcripts that encode for the coupled LcFtr1/LcFet3 proteins; orange represent constitutively expressed genes, i.e., *LcFTR1* III and *LcFRE5* I. red represents downregulated transcripts. Under iron depleted conditions, two copies of the permease (*LcFTR1* I-II) are developmentally regulated. LcFtr1 I is highly upregulated during early germination while LcFtr1 II is expressed in the germling stage. Illustrations created in Biorender (accessed on 30 December 2020).

The utilization of hemoglobin as a host molecule may also occur as putative heme oxygenases were also identified and were induced under iron limited conditions [48]. This mechanism may contribute to angioinvasion which is characteristic for systemic and deep mucormycosis [1,2,3,4]. Taken together; the RIA pathway is modelled for the human pathogenic mucoralean fungus *L. corymbifera* (Figure 8). The respective genes involved in iron uptake during early filamentous development (from swelling of the resting spores over germination to juvenile hyphenation) is primarily driven by expression of *LcFTR1* I. Our data suggest its co-expressed partner *LcFET3* I with the contribution of *LcFRE5* II and III may facilitate spore survival. Iron uptake during the later filamentous stages is supplemented by *LcFTR1* II and *LcFET3* II. The role of *LcFRE5* I, *LcFTR1* III and IV, as well as *LcFET3* III appear minor, but might be supportive under other stress conditions, which was hypothesized to be a major driving force for gene expansion in *L. corymbifera* [42]. The duplication of RIA genes and their expression as functional copies may contribute to adaption to stress tolerance and to successful manifestation of pathogenicity by supporting immune evasion, and thus escape from immune defense as reviewed for mucormycosis by [20]. Under iron starvation, *L. corymbifera* employs reductive iron assimilation genes are also upregulated [48,61].

## 5. Conclusions

The study of iron metabolism is particularly important for Mucoralean fungi as it can reveal the novel molecules that contribute to iron uptake which in turn affect virulence [130]. The active transport of ferric iron across the fungal cell membrane is mediated by dedicated high affinity iron permeases. Among those, *LcFTR1* I is the primary copy out of the four *L. corymbifera* permeases *LcFTR1* I-IV which drives iron assimilation during early filamentous development under iron starvation. The impairment of *LcFTR1* I in recognition by macrophages may be indicative for an essential role in immune evasion, and thus during manifestation of pathogenesis due to employment of macrophages for dissemination of the pathogen.

Consequently, these proteins have the potential to function as unique targets that can inhibit or block iron uptake. Therefore, they could grossly impair pathogenesis as iron acquisition is a key virulence determinant.

## Supplementary Materials

The following are available online at https://www.mdpi.com/article/10.3390/jof7040272/s1, Figure S1. Validation of the internal control genes for *L. corymbifera* FSU9682. Geometric mean of EF2α and UCE3 Ct values under experimental conditions; markers represent the mean of the geometric mean between EF2α and UCE3 of 3 biological and 2 technical replicates of each given condition; error bars indicate one SD of the Ct value for each condition; red dotted line indicates the overall mean of all samples. EF2α: Elongation factor 2 alpha; and UCE3: Ubiquitin Conjugating enzyme 3 [132]. Figure S2. Expression vectors used for heterologous expression in *S. cerevisiae FTR1* null mutants. Plasmid A: pYES2 vector was constructed for *LcFTR1* expression under a *GAL1* promoter. Plasmid B: pYES2 GAL1 promoter was replaced with the tetracycline (*TetOn*) promoter (pYES2-Tet). Both plasmids contain a *URA3* selection marker. Figure S3. Alignment of amino acid sequences of the ferric reductase of *S. cerevisiae* ScFre5 and the three copies of *L. corymbifera* ferric reductases (LcFre5 I-III). Sequences were obtained from NCBI and aligned using MAFFT in Jalview program. Protein domains: (1) Signal peptide (red box); (2) Transmembrane domain (black underline); (3) Percentage amino acid identity to ScFre5 (Purple box) were identified using the SMART online tool available from (http://smart.embl-heidelberg.de/, accessed on 1 March 2021). LcFet5 I-III; *L. corymbifera* Fre5 copies; ScFre5: *S. cerevisiae*; CaFre5: *C. albicans*; AfuFre5: *A. fumigatus*; CnFre5: *C. neofomans*. Figure S4. Alignment of amino acid sequences of ScFet3 and *L. corymbifera* LcFet3 copies. Sequences were obtained from NCBI and aligned using MAFFT in Jalview program. Illustrated are: (1) the ferroxidase motif (yellow box); (2) Copper (Cu)-ligands (dots); (3) residues involved in iron binding (Fe^2+^) binding (asterisk). Protein domains: signal peptide (red box) and transmembrane domain (underlined were identified using the SMART online tool available from (http://smart.embl-heidelberg.de/, accessed on 7 January 2021). Purple regions indicate the percentage amino acid identity. ScFet3: *S. cerevisiae*; LcFet3 (I-III); *L. corymbifera*. Figure S5. Alignment of amino acid sequences of the high affinity iron permeases Ftr1 identified in *L. corymbifera*. belonging to *S. cerevisiae* ScFtr1 and the three copies of *L. corymbifera* ferric reductases (LcFre5 I-III). Sequences were obtained from NCBI and aligned using MAFFT in Jalview program. Protein domains: (1) REGLE motif (red box) that facilitates iron binding; (2) Predicted transmembrane domain (TM) (black underline) identified for the four copies of *L. corymbifera* permease; (3) TMs identified using the online tool available from (https://harrier.nagahama-i-bio.ac.jp/sosui/sosui_submit.html, accessed on 19 March 2021). Abbreviations; ScFtr1: *S. cerevisiae*; CaFtr1: *C. albicans*; rFtr1: *R. arrhizus*; *L. corymbifera*: LcFtr1 (I-IV) for the four copies. Table S1. Strains used in this Study. Table S2. Cloning primers used heterologous expression in *S. cerevisiae.* Table S3. List of primers used in qRT-PCR analysis of *L. corymbifera*. Table S4. List of primers used for qRT-PCR in Murine alveolar macrophages (MH-S). Table S5. Iron uptake genes belonging to the Reductive pathway of *L. corymbifera.* Table S6. Expression profile analysis of *L. corymbifera* iron permease (LcFTR1 I-IV) under iron depleted conditions. Statistical comparison for FTR genes expression between various time points under iron stress. A: denotes to LcFTR1; B: represents LcFTR1 II; C shows LcFTR1 III; and D represents LcFTR1 IV. No significance: ns, * *p* < 0.05, ** *p* < 0.01, and *** *p* < 0.001. Three independent biological replicates were performed. Table S7. Expression profile analysis of *L. corymbifera* iron permease (LcFTR1 I-IV) under iron stress conditions. Statistical comparison for FTR genes expression among various time points under normal condition. A: denotes to LcFTR1; B: represents LcFTR1 II; C shows LcFTR1 III; and D represents LcFTR1 IV. No significance: ns, * *p* < 0.05, ** *p* < 0.01, and *** *p* < 0.001. Three independent biological replicates were performed.
