# Supplementary material for: Expression Patterns in Reductive Iron Assimilation and Functional Consequences during Phagocytosis of Lichtheimia corymbifera, an Emerging Cause of Mucormycosis
Source: J Fungi (Basel). 2021 Apr 3;7(4):272. doi: 10.3390/jof7040272 (PMC8065604; doi:10.3390/jof7040272)
Supplement: Supplementary file 1 [file jof-07-00272-s001.zip › jof-1124779 suppl. tables for proof.docx]

**Table S1.** Strains used in this study.

| Species | Strain | Reference |
| --- | --- | --- |
| *L. corymbifera* | FSU 09682  (CBS 429.75; JMRC, Jena, listed as WFCC no. 919 at the World Federation for Culture Collections) | [42] |
| *S. cerevisiae* (WT)^1^ | *S. cerevisiae* WT strain  BY4742 (MATα his3Δ1 leu2Δ0 lys2Δ0 ura3Δ.0) | Dharmacon-Horizon Knockout library |
| *S. cerevisiae FTR1* null mutan)^1^ | *S. cerevisiae* strain Δ*FTR1* mutant  BY4742 (MATα his3Δ1 leu2Δ0 lys2Δ0 ura3Δ.0 *FTR1*Δ::G418 | Dharmacon-Horizon Knockout library |
| *S. cerevisiae FTR1* null mutant | *S. cerevisiae* strain Δ*FTR1* mutant  BY4742 (MATα his3Δ1 leu2Δ0 lys2Δ0 ura3Δ.0 *FTR1*Δ::G418 + pYES2Tet-*LcFTR1*_I | This study |
| *S. cerevisiae FTR1* null mutant | *S. cerevisiae* strain Δ*FTR1* mutant  BY4742 (MATα his3Δ1 leu2Δ0 lys2Δ0 ura3Δ.0 *FTR1*Δ::G418 + pYES2Tet-*LcFTR1*_II | This study |
| *S. cerevisiae FTR1* null mutant | *S. cerevisiae* strain Δ*FTR1* mutant  BY4742 (MATα his3Δ1 leu2Δ0 lys2Δ0 ura3Δ.0 *FTR1*Δ::G418 + pYES2Tet-EV | This study |

^1^ Strains purchased from the Dharmacon-Horizon Company from the Yeast knockout collection.

**Table S2.** Iron uptake genes belonging to the Reductive pathway of *L. corymbifera*.

| Gene^1^ | ID^2^ | GenBank^3^ | Gene Product Name |
| --- | --- | --- | --- |
| ***LcFTR1* I**  (*FTR1* homolog) | LCor01036.1 | CDH49288 | Putative Iron Permease Copy I |
| ***LcFTR1* II**  (*FTR1* homolog) | LCor06326.1 | CDH55156 | Putative Iron Permease Copy II |
| ***LcFTR1* III**  (*FTR1* homolog) | LCor04103.1 | CDH52651 | Putative Iron Permease Copy III |
| ***LcFTR1* IV**  (*FTR1* homolog) | LCor00518.1 | CDH48747 | Putative Iron Permease Copy IV |
| ***LcFET3* I**  (FET3 homolog) | LCor01035.1 | CDH49287 | Putative Multicopper oxidase copy I |
| ***LcFET3* II**  (*FET3* homolog) | LCor06327.1 | CDH55157 | Putative Multicopper oxidase copy II |
| ***LcFET3* III**  (*FET3* homolog) | LCor04104.1 | CDH52652 | Putative Multicopper oxidase copy III |
| **Ferric reductase I**  **(*FRE5* homolog)** | LCor05212.1 | hypothetical protein | Putative Ferric reductase  copy I |
| **Ferric reductase II**  ***(FR5E* homolog)** | LCor07115.1 | CDH56027 | Putative Ferric reductase  copy II |
| **Ferric reductase III**  **(*FRE5* homolog)** | LCor11373.1 | CDH60591 | Putative Ferric reductase  copy II |

^1^Homologous sequence to *S. cerevisiae*: *FTR1*, *FET3*^2^ and *FRE5* genes respectively; ^2^Locus ID and annotation from genome database or by homology from NCBI database (http://www.ncbi.nlm.nih.gov/; *); ^3^Accession number from *L. corymbifera* obtained from the genome database; ^4^Putative protein product.

**Table S3.** List of primers used for *LcFTR1* heterologous expression in *S. cerevisiae*.

| Symbol | Description | Primers (5’-3’) |
| --- | --- | --- |
| *gFTR1* (I)  (LCor01036.1) | Putative Iron Permease Copy I  (gene specific) | F: ATGTCGCAGGATCTCTTTGATGTC  R: CTAAGCTTTATCAGTAGTGTTGTGGGG |
| *gFTR1* (II)  (LCor06326.1) | Putative Iron Permease Copy II  (gene specific) | F: CTACTTCTTTTGTTCGGTGATAGCGTC  R: ATGGCATCTCAGGACCTGTTTAACG |
| *gFTR1* (III)  (LCor04103.1) | Putative Iron Permease Copy III  (gene specific) | F: CTATTGGGTAGGCGGTGGAA  R: ATGTCTCAAGACCTCTTTTATGTTCCTATCTT |
| *gFTR1* (IV)  (LCor00518.1) | Putative Iron Permease Copy IV  (gene specific) | F: ATGGGCGGTGATCTCTTTTCGATT  R: TCAATAACGATAATGATGGGAGGATGAGAG |
| *FTR1* (I)  (LCor01036.1) | Cloning | F: AGGGAATATTAAGCTTATGTCGCAGGATCTCTTTGATGTCC  R: GATATCTGCAGAATTCTTAAGCTTTATCAGTAGTGTTGTGGGGC |
| *FTR1* (II)  (LCor06326.1) | Cloning | F: AGGGAATATTAAGCTTATGGCATCTCAGGACCTGTTTAACG  R: GATATCTGCAGAATTCTTACTTCTTTTGTTCGGTGATAGCGTCC |
| *FTR1* (III)  (LCor04103.1) | Cloning | F: AGGGAATATTAAGCTTATGTCGCAGGATCTCTTTGATGTCC  R: GATATCTGCAGAATTCTTAAGCTTTATCAGTAGTGTTGTGGGGC |
| *FTR1* (IV)  (LCor00518.1) | Cloning | F: AGGGAATATTAAGCTTATGGCATCTCAGGACCTGTTTAACG  R: GATATCTGCAGAATTCTTACTTCTTTTGTTCGGTGATAGCGTCC |
| pYES2 plasmid | - | F: CTCAACTCCATCACATCACACCGGATCGGACTACTAGCAG  R: TTTCATACACCGGGCAAAGAACTAGTGGATCATCCCCACG |
| pYES2-Tet | - | F: CTCAACTCCATCACATCACACCGGATCGGACTACTAGCAG  R: TTTCATACACCGGGCAAAGAACTAGTGGATCATCCCCACG |
| TetOn promoter | - | F: CGACGTGGTCCCTGAAGAC  R: CAGCCTTGGGAACAAGTGG |
| pYES2_677 | Sanger sequencing primers | F: GAGGAAAAATTGGCAGTAACCTGGC  R: GCGTCCCAAAACCTTCTCAAG |

**Table S4.** List of primers used in qRT-PCR analysis of *L. corymbifera*.

| Symbol | Gene Product Name | GenBank | Primers (5’-3’) | Efficiency (%) |
| --- | --- | --- | --- | --- |
| *EF2*α  (LCor01892.1) | Elongation factor *2*α | CDH50170 | F: CACGTACCATTGAAACTGCCAACG  R: GCGTTGCATCATCTTGTCTTGATCG | 95% |
| *UCE*3  (LCor09209.1) | Ubiquitin Conjugating enzyme | CDH58346 | F: TCGATCCAAGCACTGCTGTC  R: TCCTAGTCCATTCGCGTGC | 95% |
| *FTR1* I  (LCor01036.1) | Putative Iron Permease Copy I | CDH49288 | F: CCTCCCTTTCATCACTGTTC  R: GAAACCAACCAAACAACCACAG | 92% |
| *FTR1* II  (LCor06326.1) | Putative Iron Permease Copy II | CDH52651 | F: GGCGTACTGCTCAAAGAATC  R: TATGGAAAGTGCGTATGCAAC | 79% |
| *FTR1* III  (LCor04103.1) | Putative Iron Permease Copy III | CDH49287 | F: GGGAGAGTCATTCGTTTCTGTGC  R: GTGCAGGTAGAAGCCGAAAGC | 108% |
| *FTR1* IV  (LCor00518.1) | Putative Iron Permease Copy IV | CDH55157 | F: CGACGTGGTCCCTGAAGAC  R: CAGCCTTGGGAACAAGTGG | 75% |
| *FET3* I  (LCor01035.1) | Putative Multicopper oxidase copy I | CDH49287 | F: CCAGCAATGGCCATTTCATG  R: GGATGTTCCTCATGGATGGC | 90% |
| *FET3* II  (LCor06327.1) | Putative Multicopper oxidase copy II | CDH60591 | F: CGACGTGGTCCCTGAAGAC  R: CAGCCTTGGGAACAAGTGG | 80% |
| *FET3* III  (LCor04104.1) | Putative Multicopper oxidase copy III | CDH56027 | F: GCGATTGATGAATCGACACCTT  R: ATTGAGCGTGTAGCTGCAC | 81% |
| *FRE5* (I)  (LCor05212.1) | Putative Ferric reductase  copy I | CDH57122 | F: GCGTCATATTCAAAAGCGACTC  R: TGAAGGTGAACGTGTACAGC | 96% |
| *FRE5* (II)  (LCor07115.1) | Putative Ferric reductase  copy II | CDH60250 | F: CTGAGTTGCAAGCCTCTCAAG  R: GCAAGGCACGCAAAATGAC | 80% |
| *FRE5* (III)  (LCor11373.1) | Putative Ferric reductase  copy III | CDH60591 | F: GGGTCATTGGATGCCAGTTGG  R: TTTGATCGGTGTTGGCGC | 107% |
| *Ferritin* (*FER* II) (LCor08103.1) | *L. corymbifera* Ferritin copy I  (Internal iron storage) | CDH57122 | F: GCGTCATATTCAAAAGCGACTC  R: TGAAGGTGAACGTGTACAGC | 95.6% |
| *Ferritin* (*FER* II)  (LCor11038) | *L. corymbifera* Ferritin copy II  (Internal iron storage) | CDH60250 | F: CTGAGTTGCAAGCCTCTCAAG  R: GCAAGGCACGCAAAATGAC | 70.1% |

**Table S5.** List of primers used for qRT-PCR in murine alveolar macrophages (MH-S) and human macrophages (HS).

| Symbol | GenBank | Official Name | Primers (5’-3’) | Efficiency | Ref |
| --- | --- | --- | --- | --- | --- |
| *UBC* | NM_019639.4 | Ubiquitin C | F: CCAGTGTTACCACCAAGAAG  R: ACCCAAGAACAAGCACAAGG | 101% | [63], This study |
| *EEF1α1* | NM_010106 | Elongation factor 1 alpha 1 eukaryotic translation | F: GAGCCACCATACAGTCAGAAG  R: GCTACTGTGTCAGGGTTGTAG | 93% | This Study |
| *HSPA8* | BC066191 | Heat-shock protein A8 | F: TCCTCATCAAGCGCAATACC  R: GGCCCTTTCACCTTCATACA | 97% | This Study |
| *FTH1* | NM010239.2 | Ferritin heavy-chain polypeptide 1 | F: ACTGCACAAACTGGCTACTG  R: CGTGGTCACCCAGTTCTTTAAT | 87% | This study |
| *ActB* | NM_001101 | Beta-Actin | F: GGATCAGCAAGCAGGAGTATG  R: AGAAAGGGTGTAACGCAACTAA | 96% | [63], This study |
| *RPL37A* | L06499 | Ribosomal protein L37a | F: CCAAGATGAAGAGACGAGCTG  R: TGACAGCGGAAGTGGTATTG | 99% | [63], This study |
| *HSPA8* | BC016179.1 | Heat-shock protein A8 | F: ACTCCTCTTTCCCTTGGTATTG  R: TAGGTAGTGAAGGTCTGTGTCT | 90% | This study |
| *FTH1* | NM002032.3 | Ferritin heavy-chain polypeptide 1 | F: GTGCCGTTGTTCAGTTCTAATC  R: CAAGACAGCCACACCTTAGT | 90% | This study |

**Table S6.** Expression profile analysis of *L. corymbifera* iron permease (*LcFTR1 I-IV*) under iron depleted conditions. Statistical comparison for *FTR* genes expression between various time points under iron stress. A: denotes to *LcFTR1*; B: represents *LcFTR1* II; C shows *LcFTR1* III; and D represents *LcFTR1* IV. No significance: ns (non-significant). * *P<0.05*,** *P<0.01*, and *** *P<0.001*. Three independent biological replicates were performed.

|  | **A0h** | **B0h** | **C0h** | **D0h** | **A3h** | **B3h** | **C3h** | **D3h** | **A5h** | **B5h** | **C 5h** | **D 5h** | **A 8h** | **B 8h** | | **C 8h** | | **D8h** | | **A16h** | | **B16h** | | **C16h** | | **D16h** |
| --- | --- | --- | --- | --- | --- | --- | --- | --- | --- | --- | --- | --- | --- | --- | --- | --- | --- | --- | --- | --- | --- | --- | --- | --- | --- | --- |
| **A0h** |  | **ns** | **ns** | **ns** |  |  |  |  |  |  |  |  |  |  |  | |  | |  | |  | |  | |  | |
| **B0h** |  |  | ******* | **ns** |  |  |  |  |  |  |  |  |  |  |  | |  | |  | |  | |  | |  | |
| **C0h** |  |  |  | ******* |  |  |  |  |  |  |  |  |  |  |  | |  | |  | |  | |  | |  | |
| **D0h** |  |  |  |  |  |  |  |  |  |  |  |  |  |  |  | |  | |  | |  | |  | |  | |
| **A3h** |  |  |  |  |  | **ns** | **ns** | **ns** |  |  |  |  |  |  |  | |  | |  | |  | |  | |  | |
| **B3h** |  |  |  |  |  |  | ***** | ******* |  |  |  |  |  |  |  | |  | |  | |  | |  | |  | |
| **C3h** |  |  |  |  |  |  |  | **ns** |  |  |  |  |  |  |  | |  | |  | |  | |  | |  | |
| **D3h** |  |  |  |  |  |  |  |  |  |  |  |  |  |  |  | |  | |  | |  | |  | |  | |
| **A5h** |  |  |  |  |  |  |  |  |  | ****** | **ns** | **ns** |  |  |  | |  | |  | |  | |  | |  | |
| **B5h** |  |  |  |  |  |  |  |  |  |  | **ns** | **ns** |  |  |  | |  | |  | |  | |  | |  | |
| **C5h** |  |  |  |  |  |  |  |  |  |  |  | **ns** |  |  |  | |  | |  | |  | |  | |  | |
| **D5h** |  |  |  |  |  |  |  |  |  |  |  |  |  |  |  | |  | |  | |  | |  | |  | |
| **A8h** |  |  |  |  |  |  |  |  |  |  |  |  |  | **ns** | **ns** | | **ns** | |  | |  | |  | |  | |
| **B8h** |  |  |  |  |  |  |  |  |  |  |  |  |  |  | **ns** | | **ns** | |  | |  | |  | |  | |
| **C8h** |  |  |  |  |  |  |  |  |  |  |  |  |  |  |  | | **ns** | |  | |  | |  | |  | |
| **D8h** |  |  |  |  |  |  |  |  |  |  |  |  |  |  |  | |  | |  | |  | |  | |  | |
| **A16h** |  |  |  |  |  |  |  |  |  |  |  |  |  |  |  | |  | |  | | **ns** | | **ns** | | **ns** | |
| **B16h** |  |  |  |  |  |  |  |  |  |  |  |  |  |  |  | |  | |  | |  | | **ns** | | **ns** | |
| **C16h** |  |  |  |  |  |  |  |  |  |  |  |  |  |  |  | |  | |  | |  | |  | | **ns** | |
| **D16** |  |  |  |  |  |  |  |  |  |  |  |  |  |  |  | |  | |  | |  | |  | |  | |

**Table S7.** Expression profile analysis of *L. corymbifera* iron permease (*LcFTR1 I-IV*) under iron stress conditions. Statistical comparison for FTR genes expression among various time points under normal condition. A: denotes to *LcFTR1*; B: represents *LcFTR1* II; C shows *LcFTR1* III; and D represents *LcFTR1* IV. No significance: ns (non-significant). * *P<0.05*,** *P<0.01*, and *** *P<0.001*. Three independent biological replicates were performed.

|  | **A0h** | **B0h** | **C0h** | **D0h** | **A3h** | **B3h** | **C3h** | **D3h** | **A5h** | **B5h** | **C 5h** | **D 5h** | **A 8h** | **B 8h** | | **C 8h** | | **D8h** | | **A16h** | | **B16h** | | **C16h** | | **D16h** |
| --- | --- | --- | --- | --- | --- | --- | --- | --- | --- | --- | --- | --- | --- | --- | --- | --- | --- | --- | --- | --- | --- | --- | --- | --- | --- | --- |
| **A0h** |  | ***** | ***** | ***** |  |  |  |  |  |  |  |  |  |  |  | |  | |  | |  | |  | |  | |
| **B0h** |  |  | **ns** | **ns** |  |  |  |  |  |  |  |  |  |  |  | |  | |  | |  | |  | |  | |
| **C0h** |  |  |  | **ns** |  |  |  |  |  |  |  |  |  |  |  | |  | |  | |  | |  | |  | |
| **D0h** |  |  |  |  |  |  |  |  |  |  |  |  |  |  |  | |  | |  | |  | |  | |  | |
| **A3h** |  |  |  |  |  | ******* | ******* | ******* |  |  |  |  |  |  |  | |  | |  | |  | |  | |  | |
| **B3h** |  |  |  |  |  |  | ******* | ******* |  |  |  |  |  |  |  | |  | |  | |  | |  | |  | |
| **C3h** |  |  |  |  |  |  |  | **ns** |  |  |  |  |  |  |  | |  | |  | |  | |  | |  | |
| **D3h** |  |  |  |  |  |  |  |  |  |  |  |  |  |  |  | |  | |  | |  | |  | |  | |
| **A5h** |  |  |  |  |  |  |  |  |  | ******* | ******* | ******* |  |  |  | |  | |  | |  | |  | |  | |
| **B5h** |  |  |  |  |  |  |  |  |  |  | ******* | ******* |  |  |  | |  | |  | |  | |  | |  | |
| **C5h** |  |  |  |  |  |  |  |  |  |  |  | **ns** |  |  |  | |  | |  | |  | |  | |  | |
| **D5h** |  |  |  |  |  |  |  |  |  |  |  |  |  |  |  | |  | |  | |  | |  | |  | |
| **A8h** |  |  |  |  |  |  |  |  |  |  |  |  |  | ******* | ******* | | ******* | |  | |  | |  | |  | |
| **B8h** |  |  |  |  |  |  |  |  |  |  |  |  |  |  | **ns** | | **ns** | |  | |  | |  | |  | |
| **C8h** |  |  |  |  |  |  |  |  |  |  |  |  |  |  |  | | ***** | |  | |  | |  | |  | |
| **D8h** |  |  |  |  |  |  |  |  |  |  |  |  |  |  |  | |  | |  | |  | |  | |  | |
| **A16h** |  |  |  |  |  |  |  |  |  |  |  |  |  |  |  | |  | |  | | **ns** | | ******* | | ******* | |
| **B16h** |  |  |  |  |  |  |  |  |  |  |  |  |  |  |  | |  | |  | |  | | ******* | | ******* | |
| **C16h** |  |  |  |  |  |  |  |  |  |  |  |  |  |  |  | |  | |  | |  | |  | | **ns** | |
| **D16** |  |  |  |  |  |  |  |  |  |  |  |  |  |  |  | |  | |  | |  | |  | |  | |
